# Antimicrobial Sesquiterpenoid Derivatives and Monoterpenoids from the Deep-Sea Sediment-Derived Fungus *Aspergillus versicolor* SD-330

**DOI:** 10.3390/md17100563

**Published:** 2019-09-29

**Authors:** Xiao-Dong Li, Xiao-Ming Li, Xiu-Li Yin, Xin Li, Bin-Gui Wang

**Affiliations:** 1 Key Laboratory of Experimental Marine Biology, Institute of Oceanology, Chinese Academy of Sciences, Nanhai Road 7, Qingdao 266071, China; imnli@163.com (X.-D.L.); lixmqdio@126.com (X.-M.L.); 2 Yantai Institute of Coastal Zone Research, Chinese Academy of Sciences, Chunhui Road 17, Yantai 264003, China; xlyin@yic.ac.cn; 3 Laboratory of Marine Biology and Biotechnology, Qingdao National Laboratory for Marine Science and Technology, Wenhai Road 1, Qingdao 266237, China; 4 Center for Ocean Mega-Science, Chinese Academy of Sciences, Nanhai Road 7, Qingdao 266071, China

**Keywords:** deep-sea sediment-derived fungus, *Aspergillus versicolor*, bisabolane-type sesquiterpenoids, monoterpenoids, antimicrobial activity

## Abstract

Two new antimicrobial bisabolane-type sesquiterpenoid derivatives, *ent*-aspergoterpenin C (compound **1**) and 7-*O*-methylhydroxysydonic acid (**2**), and two new butyrolactone-type monoterpenoids, pestalotiolactones C (**3**) and D (**4**), along with a known monoterpenoid pestalotiolactone A (**5**) and four known bisabolane sesquiterpenoids (**6**−**9**), were isolated and identified from the deep-sea sediment-derived fungus *Aspergillus versicolor* SD-330. The structures of these compounds were elucidated on the basis of spectroscopic analysis, and the absolute configurations of the new compounds **1**−**4** were determined by the combination of NOESY and TDDFT-ECD calculations and X-ray crystallographic analysis. Additionally, we first determined and reported the absolute configuration of the known monoterpenoid pestalotiolactone A (**5**) through the X-ray crystallographic experiment. All of these isolated compounds were evaluated for antimicrobial activities against human and aquatic pathogenic bacteria. Compounds **1**, **2**, **6** and **9** exhibited selective inhibitory activities against zoonotic pathogenic bacteria such as *Escherichia coli*, *Edwardsiella tarda*, *Vibrio anguillarum* and *V. harveyi*, with MIC values ranging from 1.0 to 8.0 μg/mL.

## 1. Introduction

*Aspergillus versicolor* is a chemically brilliant fungal species with immense potential to produce a wide range of unique secondary metabolites including alkaloids [1,2,3], polyketides [4,5,6], and terpenoids [7]. Some of these metabolites exhibit intriguing biological properties, including antimicrobial properties [3,5] and cytotoxicity [6] as well as enzyme-inhibiting activities [1,4].

The bisabolane-type derivatives are a family of sesquiterpenoids which have been isolated from several biological sources, such as herb plants [8,9], marine sponges [10,11,12], and marine micro-organisms [13,14,15,16,17]. Among the attractive examples, peniciaculins A and B were isolated in 2015 from the culture of a deep sea-derived *Penicillium aculeatum* strain, SD-321, with significant antimicrobial activities [16]. In addition, aspergiterpenoid A and three related derivatives were isolated from a strain of sponge-derived fungus *Aspergillus* sp. in 2012 [17].

**Figure 1:**
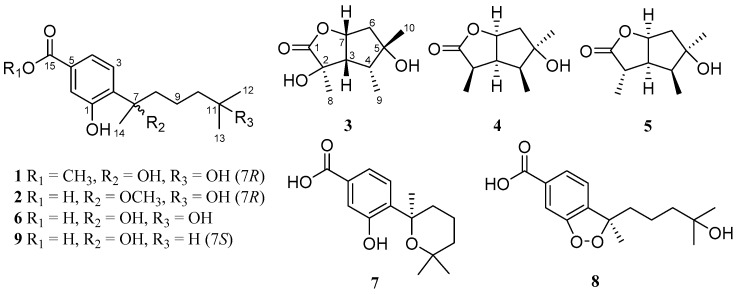
Structures of compounds **1**–**9**.

With the purpose of searching for new antibacterial metabolites from marine-derived micro-organisms, we carried out our studies to discover aromatic bisabolene-type derivatives. As a result, two new phenolic bisabolane sesquiterpenoids, *ent*-aspergoterpenin C (compound **1**) and 7-*O*-methylhydroxysydonic acid (**2**) (Figure 1), together with four known bisabolane sesquiterpenoids (**6**−**9**), as well as two new butyrolactone-type monoterpenoids, pestalotiolactones C (**3**) and D (**4**), along with a known monoterpenoid, pestalotiolactone A (**5**), were isolated and identified from the culture extract of *Aspergillus versicolor* SD-330—a deep-sea sediment-derived fungus. The structures of the isolated compounds were established by the detailed interpretation of the nuclear magnetic resonance (NMR) and mass spectrometric data, and the absolute configurations of the new compounds **1**–**4** were determined by the combination of NOESY, quantum chemical ECD calculations, and X-ray crystallographic analysis. Additionally, the absolute configuration of the known monoterpenoid compound pestalotiolactone A (**5**) was first determined and reported through the X-ray crystallographic experiment. All of these compounds were examined for antimicrobial activities against human and aquatic pathogenic bacteria. Herein, details of the isolation, structure elucidation, and biological activities of compounds **1**–**9** are described.

## 2. Results and Discussion

### 2.1. Structure Elucidation of the Compounds **1**–**5**

**Figure 2:**
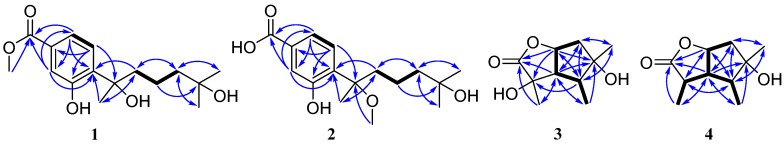
Key COSY (bold lines) and HMBC (arrows) correlations for compounds **1**–**4**.

**Figure 3:**
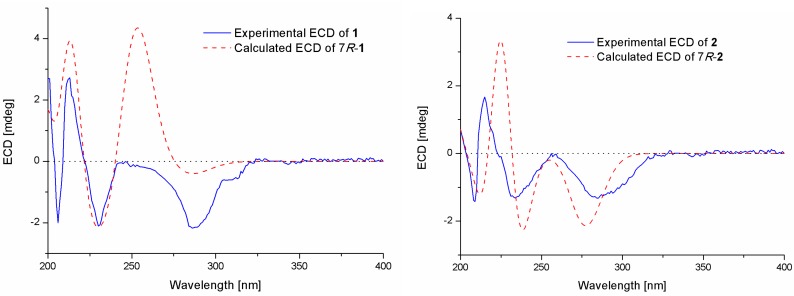
Experimental and calculated ECD spectra of compounds **1** and **2**.

Compound **1** was isolated as a colorless oil. The molecular formula was determined to be C_16_H_24_O_5_ by HRESIMS, indicating five degrees of unsaturation. The ^13^C NMR along with DEPT spectroscopic data (Table 1) revealed the presence of 16 carbon atoms, which were clarified into six non-protonated carbons, three aromatic methines, three methylenes, and four methyls (with one oxygenated). Detailed analysis of the 1D and 2D NMR spectra displayed signals similar to those of hydroxysydonic acid (compound **6**), a phenolic bisabolane sesquiterpenoid originally isolated from *Aspergillus sydowi* [18], revealing that compound **1** also belongs to the family of phenolic bisabolanes. The main differences between compounds **1** and **6** were the extra signals of a methoxy group at *δ*_H/C_ 3.80/51.8 observed in the ^1^H and ^13^C NMR spectrum of **1**. The HMBC correlation (Figure 2) from the methoxy to C-15 indicated that compound **1** is a 15-COOH methyl esterificated derivative of **6** [18]. The absolute configuration of compound **1** was determined as 7*R* based on the TDDFT-ECD calculations. The experimental ECD spectrum of compound **1** matched well with the ECD calculated for (7*R*)-**1** (Figure 3) and was opposite to the experimental ECD spectrum of aspergoterpenin C (7*S*), a bisabolane sesquiterpenoid derivative obtained from the endophytic fungus *Aspergillus versicolor* [19], revealing that compound **1** was the enantiomer of aspergoterpenin C. It is worth mentioning that aspergoterpenin C possessed a close ECD curve tp that of another related known analog, (+)-curcutetraol (7*S*) [20], suggesting that they have the same absolute configurations as 7*S*. However, the optical activities described for the two compounds were opposite ([α${]}_{D}^{20}$ = +5.24 (*c* 0.74, MeOH) for (+)-curcutetraol and [α${]}_{D}^{25}$ = −10.5 (*c* 0.07, MeOH) for aspergoterpenin C). To further confirm the absolute configuration of compound **1**, the optical rotation values of aspergoterpenin C and *ent*-aspergoterpenin C (**1**) were calculated. Combining the calculated results of aspergoterpenin C ([α${]}_{D}^{20}$ = +6.75 (*c* 0.10, MeOH)) and *ent*-aspergoterpenin C (**1**) ([α${]}_{D}^{20}$ = −6.75 (*c* 0.10, MeOH)), as well as the experimental data measured for *ent*-aspergoterpenin C (1) ([α${]}_{D}^{20}$ = −4.80 (*c* 0.40, MeOH)), we deduced that the absolute configuration of compound **1** should be assigned as 7*R*.

Compound **2** was obtained as a white amorphous powder. The molecular formula was also determined as C_16_H_24_O_5_, the same as that of compound **1**, based on HRESIMS data. The ^1^H, ^13^C, and DEPT NMR data (Table 1) of compound **2** showed identical patterns to those of compound **1**, but with some variations for the chemical shifts of C-2, C-5, C-7, C-8, C-14, and the methoxy group. The upfielded shifts of C-2 (*δ*_C_ 134.2), C-8 (*δ*_C_ 38.4), C-14 (*δ*_C_ 22.2), and oxygenated methyl (*δ*_C_ 49.4), and the downfielded shifts of C-5 (*δ*_C_ 132.2) and C-7 (*δ*_C_ 79.5) indicated that compound **2** was a 7-methoxylated, 15-COOCH_3_ demethylated derivative of compound **1**. Further evidence was observed by the key HMBC correlation from the proton of methoxyl to C-7 (Figure 2). Thus, the structure of compound **2** was determined to be 7-*O*-methylhydroxysydonic acid. The absolute configuration of compound **2** was established by the TDDFT-ECD calculation. The experimental ECD spectra of compound **2** matched well with that of calculated for (7*R*)-**2** (Figure 3), leading to the absolute configuration of compound **2** being determined as 7*R*.

To verify if compounds **1** and **2** were artifacts produced during the process of chemical isolation by the esterification or methylation of hydroxysydonic acid (compound **6**) with the methanol solvent in acidic condition, a confirmatory experiment was carried out. Hydroxysydonic acid was dissolved in MeOH with a small amount of silica gel after stirring at room temperature for 24 h. The result shows that compound **6** displayed good stability and did not react with MeOH. This was further proved by the evidence that the peaks of **1** and **2** were both detected from the high-performance liquid chromatography (HPLC) profile of crude fermented extract (Figure S15). Based on the above proofs, we tentatively presume that compounds **1** and **2** were natural products rather than artifacts.

The molecular formula of pestalotiolactone C (compound **3**) was determined to be C_10_H_16_O_4_ on the basis of HRESIMS. The spectroscopic data (Table 2) revealed the presence of 10 carbon atoms, which were clarified into three non-protonated carbons, three methines, one methylene, and three methyls. Detailed analysis of NMR data showed that the signals in ^1^H and ^13^C NMR spectra of compound **3** were similar to those of pestalotiolactone A (compound **5**), a butyrolactone-type monoterpenoid originally isolated from the axenic culture of the endophytic fungus *Pestalotiopsis* sp. [21], suggesting that compound **3** also possessed a butyrolactone-type monoterpenoid scaffold. The difference between compounds **3** and **5** was that signals of the methine (CH-2) in the 1D NMR spectra of compound **5** was absent in those of compound **3**. Instead, the presence of one oxygenated non-protonated carbon at *δ*_C_ 75.6 was observed in the ^13^C NMR spectrum of compound **3**. The above observation suggested that compound **3** was a 2-hydroxylated derivative of compound **5**. This deduction was further verified by the COSY correlations from H-6 to H-7, from H-7 to H-3, from H-3 to H-4, and from H-4 to H_3_-9 along with key HMBC correlations from H-7 and Me-8 to C-1, C-2 and C-3, from H_3_-10 to C-4, C-5 and C-6 (Figure 2). Thus, the structure of compound **3** was determined and named pestalotiolactone C.

**Figure 4:**
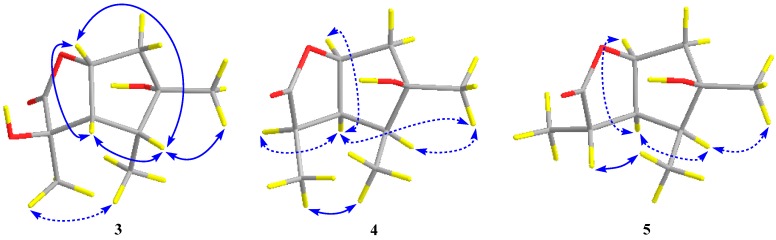
Key NOESY correlations (blue lines: *β*-orientation; blue dotted lines: *α*-orientation) for compounds **3**–**5**.

**Figure 5:**
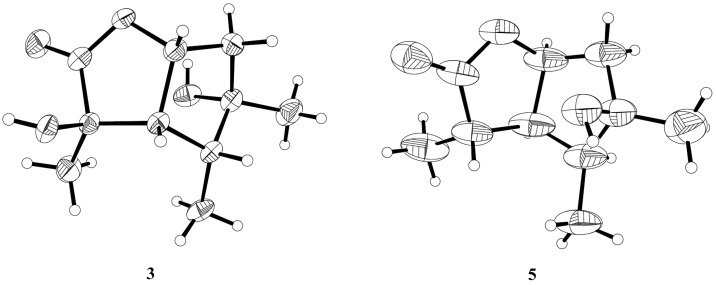
X-ray structures of compounds **3** and **5**.

The relative configuration of compound **3** was assigned by the analysis of NOESY data (Figure 4). The key NOE correlations between H-3/H-4, H-3/H-7, H-4/H-7, and H-4/Me-10 indicated that these groups were on the same side of the molecule, while the NOEs between Me-8/Me-9 suggested that they were on the other face. On the basis of the above evidence, the relative configuration of compound **3** was determined. To confirm the absolute configuration of compound **3**, we crystallized it for an X-ray single crystallographic analysis. After many attempts, single crystals that were suitable for X-ray analysis were obtained by the slow evaporation of a solution of compound **3** in CHCl_3_/MeOH = 1/9. Once the X-ray crystallographic experiment was conducted, the absolute configuration of compound 3 was unambiguously assigned as (2*R*, 3*S*, 4*R*, 5*S*, 7*R*)-**3** (Figure 5).

**Figure 6:**
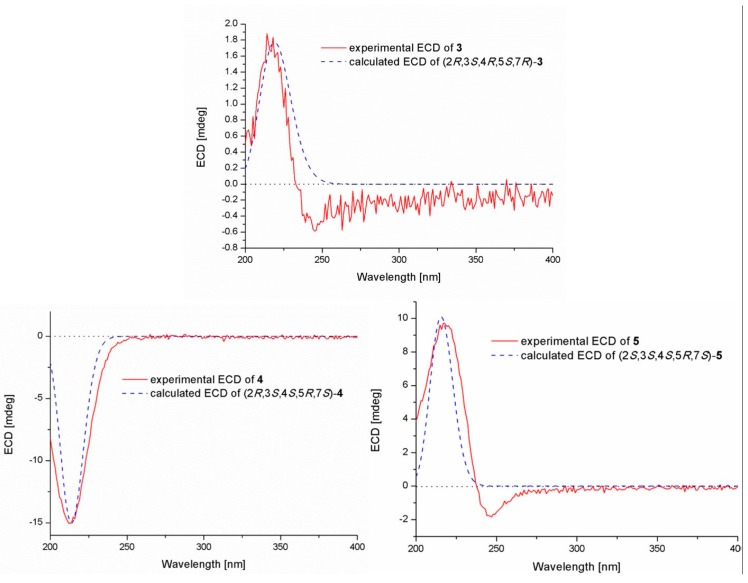
Experimental and calculated ECD spectra of compounds **3**–**5**.

Pestalotiolactone D (compound **4**) was isolated as a white amorphous powder. HRESIMS data gave the molecular formula C_10_H_16_O_3_, the same as that of pestalotiolactone A (compound **5**) [21]. The general features of the 1D NMR data (Table 2) resembled compound **5**, and a minor difference was found for the chemical shifts of H-2, H-3 and H-8 in ^1^H NMR, and C-2, C-3, C-12, and C-8 in ^13^C NMR, suggesting that compound **4** is a diastereomer of pestalotiolactone A (compound **5**). The relative configuration of **4** was assigned by analysis of NOESY data (Figure 4). The key NOE correlations between H-2/H-3, H-3/H-7, H-3/Me-10, and H-4/Me-10 indicated that these groups were on the same side of the molecule, while the NOEs between Me-8/Me-9 suggested them on the other face. On the basis of the above evidence, the relative configuration of compound **4** was determined. The absolute configuration of compound **4** was studied by the TDDFT-ECD calculation. The ECD spectrum of compound **4** exhibited negative CE at 213 nm, which matched well with that calculated for (2*R*, 3*S*, 4*S*, 5*R*, 7*S*)-**4** (Figure 6).

The structure of compound **5** was identified through the detailed analysis of ^1^H, ^13^C, and DEPT NMR data and was compared with those of pestalotiolactone A reported in the literature [21]. The relative configuration of compound **5** was determined by the key NOE correlations between H-3/H-4, H-3/H-7, H-4/Me-10 and H-7/Me-8, and the correlations between H-2/Me-9 (Figure 4), which were also same as that of pestalotiolactone A. Combined with the close optical rotation values between compound **5** and pestalotiolactone A, we finally identified the compound which was the same as pestalotiolactone A. Considering that the absolute configuration of this compound has not been reported in the literature, we crystallized it for an X-ray single crystallographic analysis. Once the X-ray crystallographic experiment was conducted, the absolute configuration of compound 5 was finally determined as (2*S*, 3*S*, 4*S*, 5*R*, 7*S*) (Figure 5). Additionally, the experimental ECD spectrum for compound 5 was also in accordance with that calculated for (2*S*, 3*S*, 4*S*, 5*R*, 7*S*)-**5** (Figure 6). This was the first report on the absolute configuration of the compound.

In addition to compounds **1**–**5**, four known bisabolane-type sesquiterpenoids (compounds **6**–**9**) were also isolated. By detailed spectroscopic analysis as well as comparisons with reported data, the structures of compounds **6**–**9** were identified as hydroxysydonic acid (compound **6**) [22], sydowic acid (compound **7**) [22], (−)-(*R*)-cyclo-hydroxysydonic acid (compound **8**) [23], and sydonic acid (compound **9**) [24].

### 2.2. Biological Activities of the Isolated Compounds

The obtained compounds **1**–**9** were tested for antimicrobial activities against seven zoonotic pathogenic bacteria (Table 3). The new compounds **1** and **2** exhibited obviously inhibitory activities against *E. coli*, *E. tarda*, *V. harveyi*, and *V. parahaemolyticus*, each with MIC values less than or equal to 8.0 μg/mL, while compound 6 exhibited significant inhibitory activity on *E*. *coli*, with an MIC value of 1.0 μg/mL, which is more potent than that of the positive control chloramphenicol (MIC 2.0 μg/mL). Moreover, compound 6 also exhibited potent activities against *Aeromonas hydrophilia*, *E. tarda*, *V. anguillarum* and *V. harveyi* each with an MIC value of 4.0 μg/mL, which is comparable to that of chloramphenicol. Compound 6 was more active toward bacteria than compounds **1** and **2**, suggesting that the C-15 carboxy group methyl ester or the methylated C-7 hydroxy group are decreasing its antimicrobial activity. The fact that compound 6 was also more active than compounds **7** and **8** indicated that the formation of cyclohexanol ether or a peroxide ring in the molecule would also decrease its activity. In addition to the bisabolane-type sesquiterpenoids, the butyrolactone-type monoterpenoids—compounds **3**–**5**—also exhibited some selective activities against the zoonotic pathogenic bacteria.

## 3. Experimental Section

### 3.1. General Experimental Procedures

Optical rotations were acquired on an Optical Activity AA-55 polarimeter (Optical Activity Ltd., Cambridgeshire, UK). UV spectra were measured on a PuXi TU-1810 UV−visible spectrophotometer (Shanghai Lengguang Technology Co., Ltd., Shanghai, China). ECD spectra were measured on a Chirascan spectropolarimeter (Applied Photophysics Ltd., Surrey, UK). One-dimensional and 2D NMR spectra were obtained at 500 and 125 MHz for 1H and 13C, respectively, on a Bruker Avance 500 MHz spectrometer (Bruker Biospin Group, Karlsruhe, Germany) with TMS as the internal standard. Mass spectra were generated on a VG Autospec 3000 (VG Instruments, London, UK) or an API QSTAR Pulsar 1 mass spectrometer (Applied Biosystems, Foster, Waltham, MA, USA). Analytical and semi-preparative HPLC procedures were performed using a Dionex HPLC system (Dionex, Sunnyvale, CA, USA) equipped with a P680 pump, an ASI-100 automated sample injector, and a UVD340U multiple wavelength detector controlled by Chromeleon software (version 6.80). Commercially available Si gel (200−300 mesh, Qingdao Haiyang Chemical Co., Qingdao, China), Lobar LiChroprep RP-18 (40−63 μm, Merck, Darmstadt, Germany), and Sephadex LH-20 (Pharmacia, Pittsburgh, PA, USA) were used for open column chromatography. All solvents were distilled prior to use.

### 3.2. Fungal Material

The fungus *Aspergillus versicolor* SD-330 was isolated from a marine sediment sample collected in May 2012 from the South China Sea at a depth of 1487 m. The fungus was identified using a molecular biological protocol by DNA amplification and the sequencing of the intrnal transcribed spacer (ITS) region, as described in our previous report [25]. The sequenced data derived from the fungal strain have been deposited in GenBank (accession no. MN176407). A BLAST search result showed that the sequence was most similar (99%) to the sequence of *Aspergillus versicolor* (compared to accession no. MH911415.1). The strain is preserved at the Key Laboratory of Experimental Marine Biology, Institute of Oceanology, Chinese Academy of Sciences, with accession number SD-330.

### 3.3. Fermentation

For chemical investigations, the fungal strain was statically fermented for 35 days at room temperature in a liquid medium containing 20% potato juice, 2% glucose, 0.5% peptone, and 0.3% yeast extract.

### 3.4. Extraction and Isolation

The entire fermented cultures were filtered to separate the broth from the mycelia. The broth was extracted three times with EtOAc, while the mycelia was extracted three times with a mixture of ethyl alcohol and H_2_O (95:5, v/v). The ethyl alcohol solution was evaporated under reduced pressure to afford an aqueous solution, which was then extracted with EtOAc three times. Because the TLC and HPLC profiles of the two EtOAc solutions from the broth and mycelia were almost identical, they were combined and concentrated under reduced pressure to give an extract (25.1 g) for further separation.

The organic extract was fractionated by vacuum liquid chromatography (VLC) on silica gel eluting with different solvents of increasing polarity from petroleum ether (PE), to MeOH to yield 9 fractions (Frs. 1–9) that were pooled based on TLC analysis. Fr. 4 (4.5 g), eluted with PE–EtOAc (2:1), was further purified by column chromatography (CC) on Sephadex LH-20 (MeOH) to afford compound 5 (15.8 mg). Fr. 5 (3.7 g), eluted with CHCl_3_–MeOH (20:1), was further purified by CC on silica gel, eluting with a PE–EtOAc gradient (from 5:1 to 2:1), to afford two subfractions (Fr. 5-1 and Fr. 5-2). Fr. 5-1 was further purified by CC on Sephadex LH-20 (MeOH) and then purified by semi-preparative HPLC (75% MeOH–H_2_O, 5 mL/min) to afford compounds **3** (15.4 mg, *t*_R_ 25.4 min) and 4 (10.7 mg, *t*_R_ 28.2 min). Fr. 5-2 was further purified by CC over RP-18 eluting with a MeOH–H_2_O gradient (from 1:9 to 1:0) and by CC on Sephadex LH-20 (MeOH) to afford compounds 7 (8.7 mg, *t*_R_ 19.2 min) and 8 (18.8 mg, *t*_R_ 20.5 min). Fr. 6 (2.1 g), eluted with CHCl_3_–MeOH (10:1), was purified by CC on silica gel eluting with a CHCl_3_–MeOH gradient (40:1 to 10:1) to afford two subfrations (Fr. 6-1 and Fr. 6-2). Fr. 6-1 was further purified by CC over RP-18 eluting with a MeOH–H_2_O gradient (1:9 to 1:0) and by semi-preparative HPLC (MeOH/H_2_O, 80% to 85%, 5 mL/min) to obtain compound 6 (30.0 mg, *t*_R_ 18.8 min). Fr. 6-2 was further purified by Sephadex LH-20 (MeOH) and by semi-preparative HPLC (80% MeOH/H_2_O, 5 mL/min) to yield compound 9 (51.5 mg, *t*_R_ 18.2 min). Fr. 7 (3.7 g), eluted with CHCl_3_–MeOH (5:1), was further purified by CC on Sephadex LH-20 (MeOH) and then purified by semi-preparative HPLC (80% MeOH–H_2_O, 5 mL/min) to obtain compounds **1** (10.9 mg, *t*_R_ 20.7 min) and **2** (18.8 mg, *t*_R_ 22.3 min).

*ent*-Aspergoterpenin C (**1**): Colorless oily liquid; [α${]}_{D}^{20}$ = −4.8 (*c* 0.40, MeOH); UV (MeOH) *λ*_max_ (log *ε*) 215 (1.86), 248 (0.62), 303 (0.20) nm; ECD (0.26 mg/mL, MeOH) *λ*_max_ (Δ*ε*) 206 (−0.68), 213 (+0.93), 231 (−0.71), 289 (−0.74) nm; ^1^H and ^13^C NMR data, Table 1 and Table 2; HRESIMS *m/z* 314.1960 [M + NH_4_]^+^ (calcd for C_16_H_28_O_5_N, 314.1962, Δ −0.5871 ppm), 319.1520 [M + Na]^+^ (calcd for C_16_H_24_O_5_Na, 319.1516, Δ 1.3681 ppm).

7-*O*-Methylhydroxysydonic acid (**2**): Amorphous powder; [α${]}_{D}^{20}$ = −4.2 (*c* 0.35, MeOH); UV (MeOH) *λ*_max_ (log *ε*) 212 (3.40), 245 (0.94), 301 (0.33) nm; ECD (0.24 mg/mL, MeOH) *λ*_max_ (Δ*ε*) 209 (−0.53), 215 (+0.62), 235 (−0.47), 289 (−0.46) nm; ^1^H and ^13^C NMR data, Table 1 and Table 2; HRESIMS *m/z* 319.1514 [M + Na]^+^ (calcd for C_16_H_24_O_5_Na, 319.1516, Δ −0.4571 ppm).

Pestalotiolactones C (**3**): Colorless single crystal (MeOH); mp 103−106 °C; [α${]}_{D}^{20}$ −17.5 (*c* 0.59, MeOH); ECD (1.1 mg/mL, MeOH) *λ*_max_ (Δ*ε*) 214 (+0.10), 246 (−0.03) nm; ^1^H and ^13^C NMR data, Table 1 and Table 2; HRESIMS *m/z* 201.1121 [M + H]^+^ (calcd for C_10_H_17_O_4_, 201.1121, Δ −0.3112 ppm), 218.1388 [M + NH_4_]^+^ (calcd for C_10_H_20_O_4_N, 218.1387, Δ 0.3683 ppm), 223.0942 [M + Na]^+^ (calcd for C_10_H_16_O_4_Na, 223.0941, Δ 0.5540 ppm).

Pestalotiolactones D (**4**): Amorphous powder; [α${]}_{D}^{20}$ −20.3 (*c* 0.58, MeOH); ECD (1.0 mg/mL, MeOH) *λ*_max_ (Δ*ε*) 213 (−0.84) nm; ^1^H and ^13^C NMR data, Table 1 and Table 2; HRESIMS *m/z* 185.1169 [M + H]^+^ (calcd for C_10_H_17_O_3_, 185.1172, Δ −1.8331 ppm), 202.1435 [M + NH_4_]^+^ (calcd for C_10_H_20_O_3_N, 202.1438, Δ −1.3100 ppm).

Pestalotiolactones A (**5**): Colorless single crystal (MeOH); mp 104−107 °C; [α${]}_{D}^{20}$ −30.3 (*c* 0.97, MeOH); ECD (1.40 mg/mL, MeOH) *λ*_max_ (Δ*ε*) 220 (+0.55), 247 (−0.11) nm; HRESIMS *m/z* 185.1172 [M + H]^+^ (calcd for C_10_H_17_O_3_, 185.1172, Δ −0.1762 ppm), 202.1438 [M + NH_4_]^+^ (calcd for C_10_H_20_O_3_N, 202.1438, Δ −0.0112 ppm), 207.0991 [M + Na]^+^ (calcd for C_10_H_16_O_3_Na, 207.0992, Δ −0.4691 ppm).

### 3.5. Antimicrobial Assays

An antimicrobial evaluation against seven zoonotic pathogenic bacteria between humans and aquatic animals (*E. coli* QDIO-1, *Aeromonas hydrophilia* QDIO-3, *E. tarda* QDIO-4, *Pseudomonas aeruginosa* QDIO-6, *V. anguillarum* QDIO-8, *V. harveyi* QDIO-9, and *V. parahaemolyticus* QDIO-10) was carried out by the microplate assay with three repetitions [26]. For each organism, a loopful of glycerol stock was streaked on a Luria Broth (LB)-agar plate, which was incubated 24 h at 37 °C. A single bacterial colony was picked and suspended in Mueller–Hinton broth to approximately 5 × 10^5^ cfu/mL. A two-fold serial dilution of each compound to be tested (2560 to 2.5 µg/mL in DMSO) was prepared and an aliquot of each dilution (5 μL) was added to a 96-well flat-bottom microtiter plate. An aliquot (95 μL) of bacterial suspension was then added to each well (to give final compound concentrations of 128 to 0.125 μg/mL in 2.0% DMSO) and the plate was incubated at 37 °C aerobically for 24 h. Finally, the optical density of each well at 600 nm was measured with an Tecan GENios multifunctional microplate reader (infinite M1000 PRO, Männedorf, Switzerland). MIC values were defined as the minimum concentration of compound that inhibited visible bacterial growth. The pathogenic bacteria and aquatic pathogen strains were provided by the Institute of Oceanology, Chinese Academy of Sciences. Chloramphenicol was used as a positive control, and DMSO as the negative control.

### 3.6. X-ray Crystallographic Analysis

Colorless crystals of compounds 3 and 5 were obtained by the slow evaporation of a solution in CHCl_3_/MeOH = 1/9, and from a solution of MeOH, respectively. Crystallographic data were collected on a Srigaku Mercury CCD/AFCR diffractometer equipped each with graphite-monochromatic Cu Kα radiation (λ = 1.54178 Å) at 293(2) K [27]. The data were corrected for absorption by using the program SADABS [28]. The structure was solved by direct methods and subsequent difference Fourier synthesis and refined by full-matrix least-squares techniques with the SHELXTL software package [29]. All non-hydrogen atoms were refined anisotropically. The H atoms belonging to C atoms were calculated theoretically, and those to O atoms were determined by difference Fourier maps [30].

Crystal data of compound **3**: C_10_H_16_O_4_; fw = 200.23, Orthorhombic space group *P2(1)2(1)2(1)*, unit cell dimensions *a* = 6.4948(6) Å, *b* = 6.6256(5) Å, *c* = 23.926(2) Å, *V* = 1029.57(16) Å^3^, *α* = 90.00, *β* = 90.00, *γ* = 90.00, *Z* = 4, *d*_calcd_ = 1.292 mg/m^3^, crystal dimensions 0.37 × 0.18 × 0.10 mm, *μ* = 0.826 mm^−1^, *F*(000) = 432. The 1452 measurements yielded 1067 independent reflections after equivalent data were averaged, and Lorentz and polarization corrections were applied. The final refinement gave *R*_1_ = 0.0283 and *wR_2_* = 0.0585 [*I* > 2*σ*(*I*)]. The Flack parameter was 0.0(5) in the final refinement for all 1452 reflections with 1067 Friedel pairs.

Crystal data of compound **5**: C_10_H_16_O_3_; fw = 184.23, Orthorhombic space group *P2(1)2(1)2(1)*, unit cell dimensions *a* = 9.3551(8) Å, *b* = 9.6715(15) Å, *c* = 11.2278(8) Å, *V* = 1015.87(19) Å^3^, *α* = 90.00, *β* = 90.00, *γ* = 90.00, *Z* = 4, *d*_calcd_ = 1.205 mg/m^3^, crystal dimensions 0.23 × 0.14 × 0.08 mm, *μ* = 0.717 mm^−1^, *F*(000) = 400. The 1544 measurements yielded 786 independent reflections after equivalent data were averaged, and Lorentz and polarization corrections were applied. The final refinement gave *R*_1_ = 0.0337 and *wR_2_* = 0.0708 [*I* > 2*σ*(*I*)]. The Flack parameter was 0.0(9) in the final refinement for all 1544 reflections with 786 Friedel pairs.

### 3.7. Computational Section

Conformational searches were performed via molecular mechanics using the MM+ method in HyperChem 8.0 software, and the geometries were further optimized at B3LYP/6-31G(d) PCM/MeCN level via Gaussian 09 software [31] to give the energy-minimized conformers. ECD and optical rotation (OR) calculations were performed with Gaussian 09. After this, the optimized conformers were subjected to the calculations of ECD spectra using TDDFT at PBE0/TZVP; solvent effects of the MeCN solution were evaluated at the same DFT level using the SCRF/Polarizable Continuum Model (PCM) method. OR values were computed using functional PBE0 and the TZVP basis set.

## 4. Conclusions

In summary, we have isolated and characterized two new antimicrobial bisabolane sesquiterpenoid derivatives, *ent*-aspergoterpenin C (compound **1**) and 7-*O*-methylhydroxysydonic acid (**2**), and two new butyrolactone-type monoterpenoids, pestalotiolactones C (**3**) and D (**4**), along with a known monoterpenoid, pestalotiolactone A (**5**), and four known bisabolene sesquiterpenoids (**6**−**9**) from the deep-sea sediment-derived fungus *Aspergillus versicolor* SD-330. The new phenolic bisabolane derivatives, compounds **1** and **2**, and the known phenolic bisabolane sesquiterpenoid, compound **6**, may prove useful as antibacterial agents since they constitute potent antimicrobial activities against zoonotic pathogenic bacteria between humans and aquatic animals.

## Figures and Tables

**Table 1:** ^1^H and ^13^C data of compounds **1** and **2** (measured in DMSO-*d*_6_).

No.	1		2	
	*δ*_H_ (Mult, *J* in Hz) ^a^	*δ*_C_, Type ^b^	*δ*_H_ (Mult, *J* in Hz) ^a^	*δ*_C_, Type ^b^
1	-	155.0, qC	-	154.4, qC
2	-	138.6, qC	-	134.2, qC
3	7.44, d (8.1)	127.1, CH	7.30, d (8.0)	127.3, CH
4	7.34, dd (8.1, 1.6)	119.2, CH	7.35, d (8.0)	119.7, CH
5	-	128.7, qC	-	132.2, qC
6	7.31, d (1.6)	116.4, CH	7.36, s	116.9, CH
7	-	74.6, qC	-	79.5, qC
8a	1.93, dt (13.0, 4.4)	41.8, CH_2_	1.87, t (11.4)	38.4, CH_2_
8b	1.65, dt (13.0, 4.0)	-	1.76, t (11.4)	-
9a	1.30, m	18.5, CH_2_	1.19, m	18.2, CH_2_
9b	1.02, m	-	1.03, m	-
10	1.23, m	44.0, CH_2_	1.22, t (12.3)	43.8, CH_2_
11	-	68.7, qC	-	68.6, qC
12	0.97, s	29.3, CH_3_	0.97, s	29.2, CH_3_
13	0.95, s	29.2, CH_3_	0.97, s	29.2, CH_3_
14	1.50, s	28.1, CH_3_	1.54, s	22.2, CH_3_
15	-	166.2, qC	-	167.9, qC
15-COOCH_3_	3.80, s	51.8, CH_3_	-	-
7-OCH_3_	-	-	3.15, s	49.4, CH_3_

^a^ Measured at 500 MHz; ^b^ Measured at 125 MHz.

**Table 2:** ^1^H and ^13^C data of compounds **3** and **4** (measured in DMSO-*d*_6_).

No.	3		4	
	*δ*_H_ (Mult, *J* in Hz) ^a^	*δ*_C_, Type ^b^	*δ*_H_ (Mult, *J* in Hz) ^a^	*δ*_C_, Type ^b^
1	-	178.1, qC	-	179.5, qC
2	-	75.6, qC	2.89, m	38.2, CH
3	2.72, dd (8.9, 7.3)	53.9, CH	2.99, m	44.3, CH
4	1.84, m	46.0, CH	1.78, m	46.0, CH
5	-	77.5, qC	-	77.8, qC
6α	1.91, d (14.3)	45.5, CH_2_	1.92, d (14.3)	45.7, CH_2_
6β	1.73, dd (14.3, 6.5)	-	1.81, dd (14.3, 6.9)	-
7	4.93, t (6.8)	80.3, CH	4.80, t (6.7)	81.0, CH
8	1.39, s	21.6, CH_3_	1.22, d (7.3)	12.3, CH_3_
9	1.02, d (7.5)	9.7, CH_3_	0.98, d (7.5)	10.2, CH_3_
10	1.08, s	24.8, CH_3_	1.11, s	26.0, CH_3_

^a^ Measured at 500 MHz; ^b^ Measured at 125 MHz.

**Table 3:** Antimicrobial activities of compounds **1**–**9** (MIC, μg/mL) ^a^.

Strains	1	2	3	4	5	6	7	8	9	Positive Control
*Escherichia coli* ^b^	4.0	2.0	–	–	–	1.0	–	32	16	2.0
*Aeromonas hydrophilia* ^b^	–	8.0	16	–	–	4.0	16	32	8.0	2.0
*Edwardsiella tarda* ^b^	8.0	4.0	–	32	–	4.0	32	–	8.0	2.0
*Pseudomonas aeruginosa* ^b^	8.0	32	–	–	–	8.0	–	–	–	2.0
*Vibrio anguillarum* ^b^	–	4.0	32	–	32	4.0	32	32	32	0.5
*Vibrio harveyi* ^b^	8.0	8.0	–	32	32	4.0	16	32	16	4.0
*Vibrio parahaemolyticus* ^b^	8.0	8.0	16	–	–	8.0	–	–	32	1.0

^a^ (−) = MIC > 32 μg/mL, ^b^ Chloramphenicol as positive control.

## Supplementary Materials

The following are available online at https://www.mdpi.com/1660-3397/17/10/563/s1, HRESIMS, 1D and 2D NMR spectra, UVs and ECDs of compounds **1**–**5**.
