# Supplementary material for: Antimicrobial Sesquiterpenoid Derivatives and Monoterpenoids from the Deep-Sea Sediment-Derived Fungus Aspergillus versicolor SD-330
Source: Mar Drugs. 2019 Sep 29;17(10):563. doi: 10.3390/md17100563 (PMC6836274; doi:10.3390/md17100563)
Supplement: Supplementary file 1 [file marinedrugs-17-00563-s001.pdf]

Supplementary File

for

Antimicrobial Sesquiterpenoid Derivatives and Monoterpenoids from the Deep-Sea Sediment-Derived Fungus *Aspergillus versicolor* SD-330

Xiao-Dong Li [1,3], Xiao-Ming Li [1,2,4], Xiu-Li Yin [3], Xin Li [1,2,4,*] and Bin-Gui Wang [1,2,4,*]

[1] Key Laboratory of Experimental Marine Biology, Institute of Oceanology, Chinese Academy of Sciences, Nanhai Road 7, Qingdao 266071, China; E-Mails: imnli@163.com (X.-D.L.); lixmqdio@126.com (X.-M.L.)

[2] Laboratory of Marine Biology and Biotechnology, Qingdao National Laboratory for Marine Science and Technology, Wenhai Road 1, Qingdao 266237, China

[3] Yantai Institute of Coastal Zone Research, Chinese Academy of Sciences, Chunhui Road 17, Yantai 264003, China; E-Mails: xlyin@yic.ac.cn (X.-L.Y.)

[4] Center for Ocean Mega-Science, Chinese Academy of Sciences, Nanhai Road 7, Qingdao 266071, China

* Correspondence: lixin@qdio.ac.cn (X.L.); wangbg@ms.qdio.ac.cn (B.-G.W.); Tel.: +86-532-8289 8553 (B.-G.W.)

Content

Figure S1. HRESI mass spectrum of compound **1**.

Figure S2. ^{1}H NMR (500 MHz, DMSO-d_6) spectrum of compound **1**.

Figure S3. ^{13}C NMR (125 MHz, DMSO-d_6) and DEPT spectra of compound **1**.

Figure S4. COSY (DMSO-d_6) spectrum of compound **1**.

Figure S5. HSQC (DMSO-d_6) spectrum of compound **1**.

Figure S6. HMBC (DMSO-d_6) spectrum of compound **1**.

Figure S7. ECD and UV spectra of compound **1**.

Figure S8. HRESI mass spectrum of compound **2**.

Figure S9. ^{1}H NMR (500 MHz, DMSO-d_6) spectrum of compound **2**.

Figure S10. ^{13}C NMR (125 MHz, DMSO-d_6) and DEPT spectra of compound **2**.

Figure S11. COSY (DMSO-d_6) spectrum of compound **2**.

Figure S12. HSQC (DMSO-d_6) spectrum of compound **2**.

Figure S13. HMBC (DMSO-d_6) spectrum of compound **2**.

Figure S14. ECD and UV spectra of compound **2**.

Figure S15. HPLC profile of the crude fermented extract.

Figure S16. HRESI mass spectrum of compound **3**.

Figure S17. ^{1}H NMR (500 MHz, DMSO-d_6) spectrum of compound **3**.

Figure S18. ^{13}C NMR (125 MHz, DMSO-d_6) and DEPT spectra of compound **3**.

Figure S19. COSY (DMSO-d_6) spectrum of compound **3**.

Figure S20. HSQC (DMSO-d_6) spectrum of compound **3**.

Figure S21. HMBC (DMSO-d_6) spectrum of compound **3**.

Figure S22. NOESY (DMSO-d_6) spectrum of compound **3**.

Figure S23. ECD and UV spectra of compound **3**.

Figure S24. HRESI mass spectrum of compound **4**.

Figure S25. ^{1}H NMR (500 MHz, DMSO-d_6) spectrum of compound **4**.

Figure S26. ^{13}C NMR (125 MHz, DMSO-d_6) and DEPT spectra of compound **4**.

Figure S27. COSY (DMSO-d_6) spectrum of compound **4**.

Figure S28. HSQC (DMSO-d_6) spectrum of compound **4**.

Figure S29. HMBC (DMSO-d_6) spectrum of compound **4**.

Figure S30. NOESY (DMSO-d_6) spectrum of compound **4**.

Figure S31. ECD and UV spectra of compound **4**.

Figure S32. ^{1}H NMR (500 MHz, DMSO-d_6) spectrum of compound **5**.

Figure S33. ^{13}C NMR (125 MHz, DMSO-d_6) and DEPT spectra of compound **5**.

Figure S34. NOESY (DMSO-d_6) spectrum of compound **5**.

Figure S35. ECD and UV spectra of compound **5**.

Table S1. Abbreviation list.

Figure S1. HRESI mass spectrum of compound **1**.

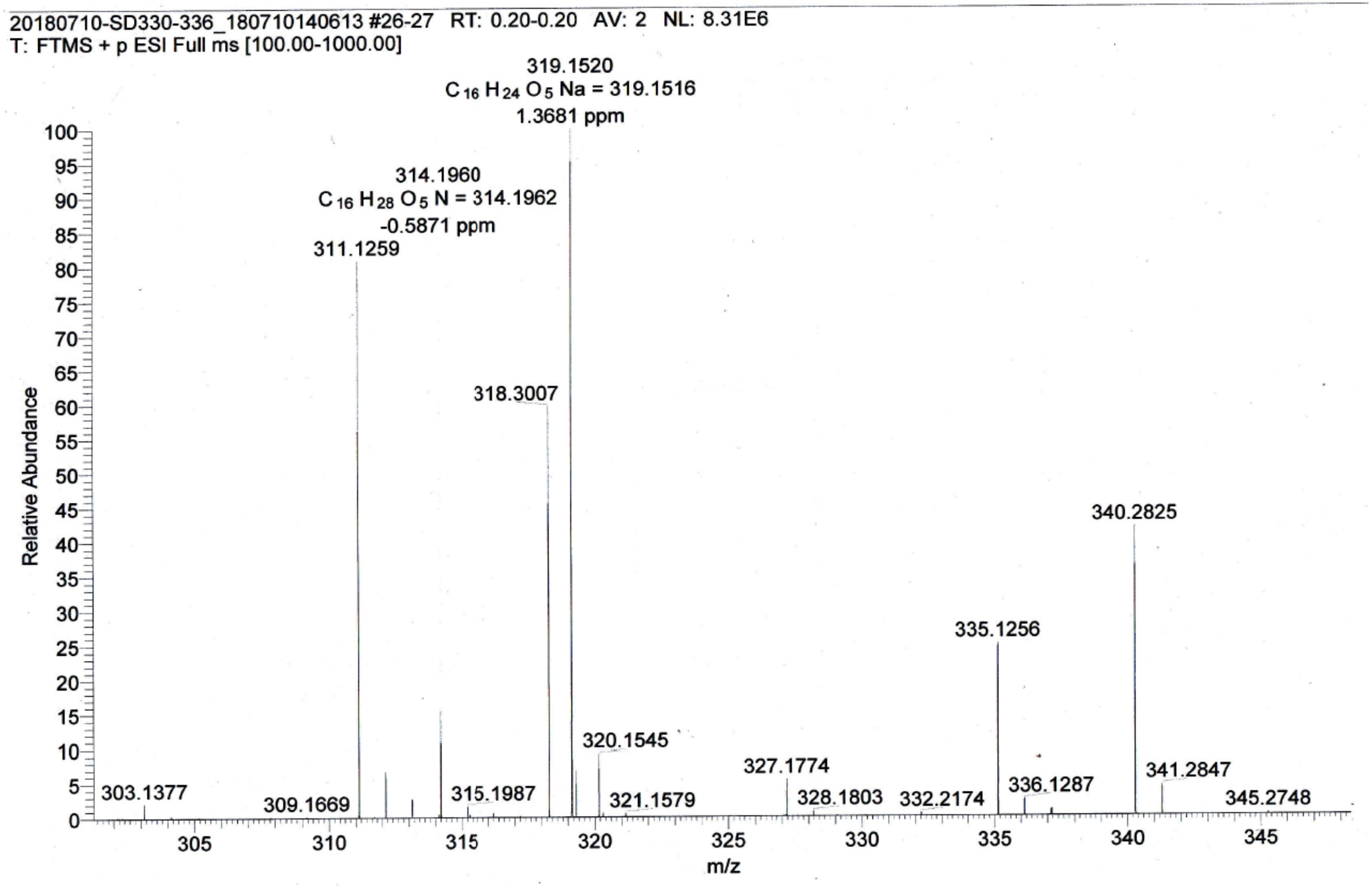

Figure S2. ^{1}H NMR (500 MHz, DMSO-d_6) spectrum of compound **1**.

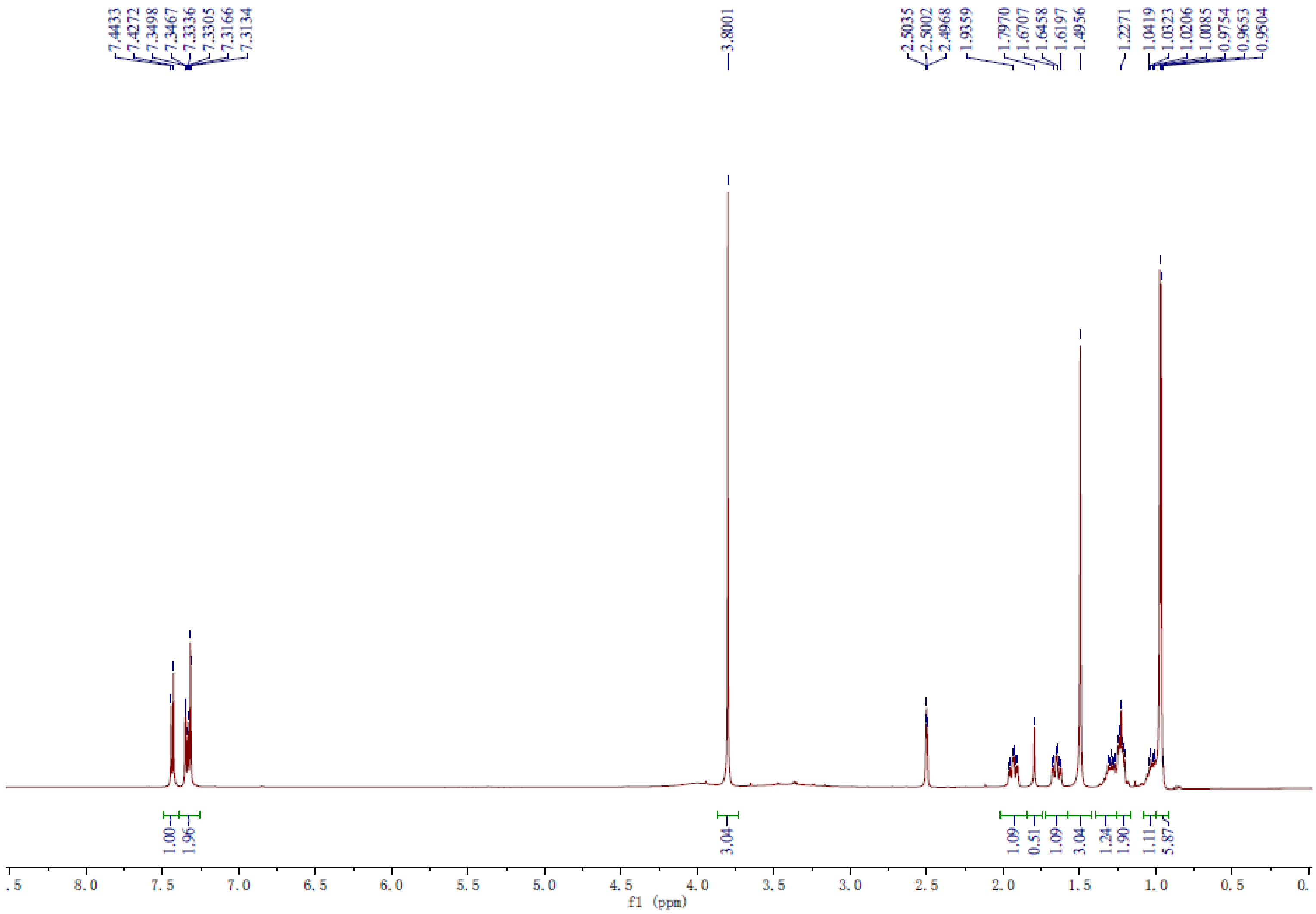

Figure S3. ^{13}C NMR (125 MHz, DMSO-d_6) and DEPT spectra of compound **1**.

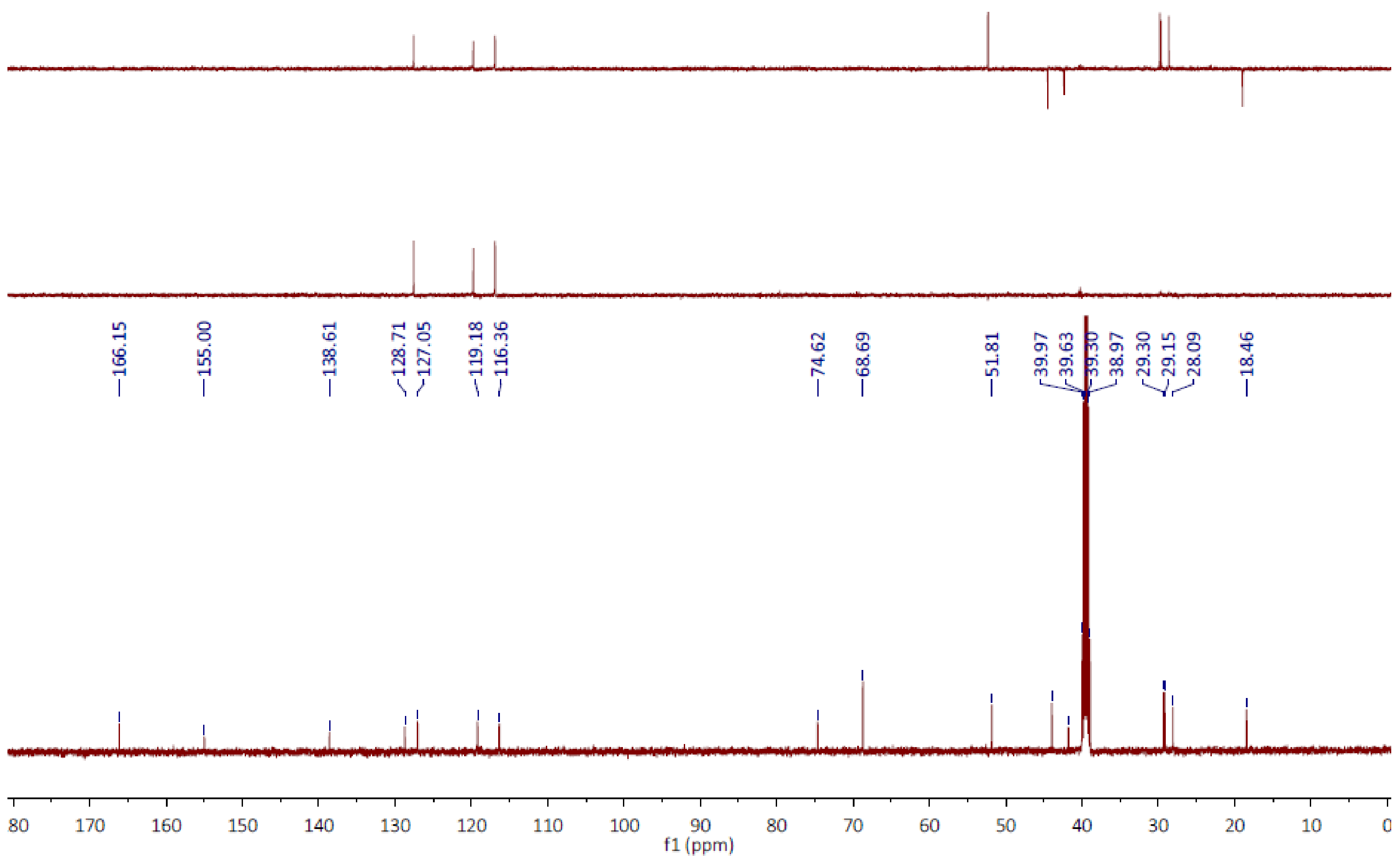

Figure S4. COSY (DMSO-d_6) spectrum of compound **1**.

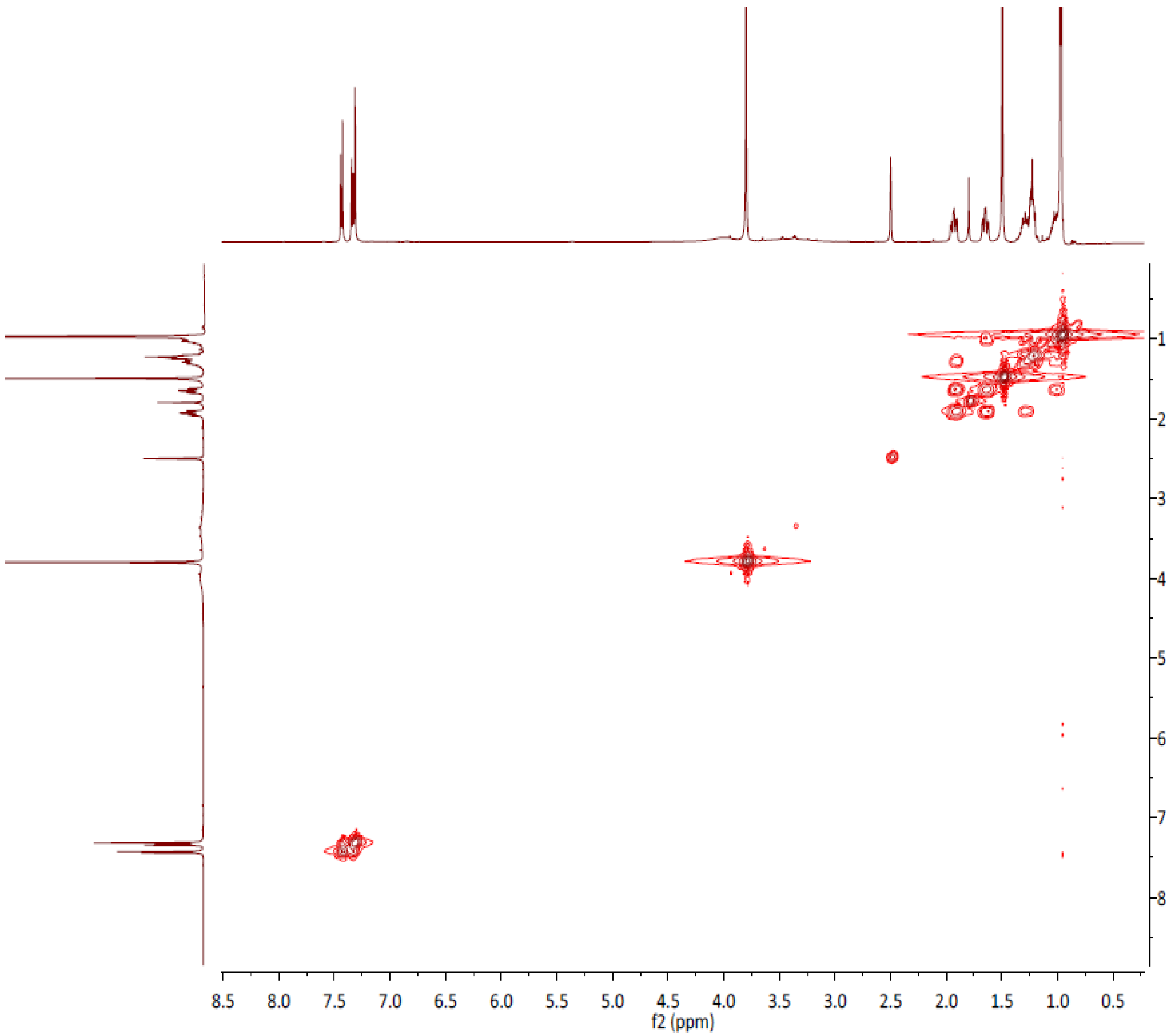

Parameters		
	Parameter	Value (f2, f1)
1	Owner	psm
2	Spectrometer	av500
3	Solvent	
4	Temperature	296.4
5	Pulse Sequence	cosygpqf
6	Number of Scans	4
7	Receiver Gain	64
8	Relaxation Delay	1.4869
9	Pulse Width	10.0000
10	Acquisition Time	0.0788
11	Acquisition Date	2018-05-04T16:21:54
12	Modification Date	2018-05-04T16:49:02
13	G to G'	1.00000000
14	Echo Delay	0.0000
15	Gradient Duration	0.0000
16	Spectrometer Frequency	(499.80, 499.80)
17	Spectral Width	(6510.4, 6497.2)
18	Lowest Frequency	(-261.4, -254.8)
19	Nucleus	(1H, 1H)
20	Acquired Size	(512, 256)
21	Spectral Size	(512, 512)

Figure S5. HSQC (DMSO-d_6) spectrum of compound **1**.

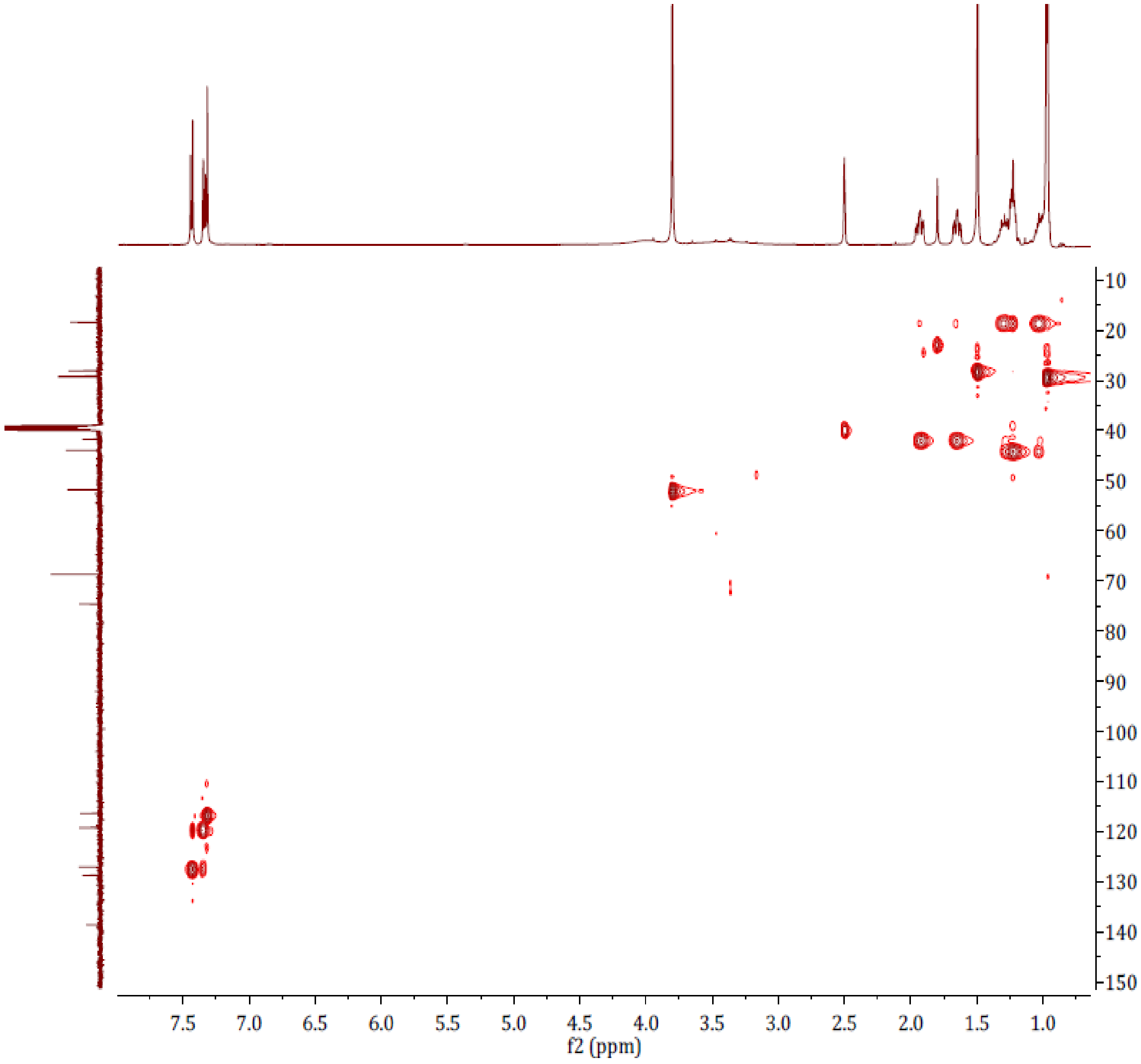

	Parameter	Value
1	Title	360$6
2	Comment	HSQC
3	Origin	Bruker BioSpin GmbH
4	Owner	nmr600
5	Spectrometer	spect
6	Solvent	DMSO
7	Temperature	298.0
8	Pulse Sequence	hsqcetgpsisp2.2
9	Number of Scans	2
10	Receiver Gain	181
11	Relaxation Delay	1.5000
12	Pulse Width	7.9000
13	Acquisition Time	0.0940
14	Acquisition Date	2018-11-30T16:52:41
15	Modification Date	2018-11-30T17:03:29
16	G to G'	1.00000000
17	Echo Delay	0.0000
18	Gradient Duration	0.0017
19	Spectrometer Frequency	600.13
20	Spectral Width	5446.6
21	Lowest Frequency	-61.7
22	Nucleus	1H
23	Acquired Size	512
24	Spectral Size	1024

Figure S6. HMBC (DMSO-d_6) spectrum of compound **1**.

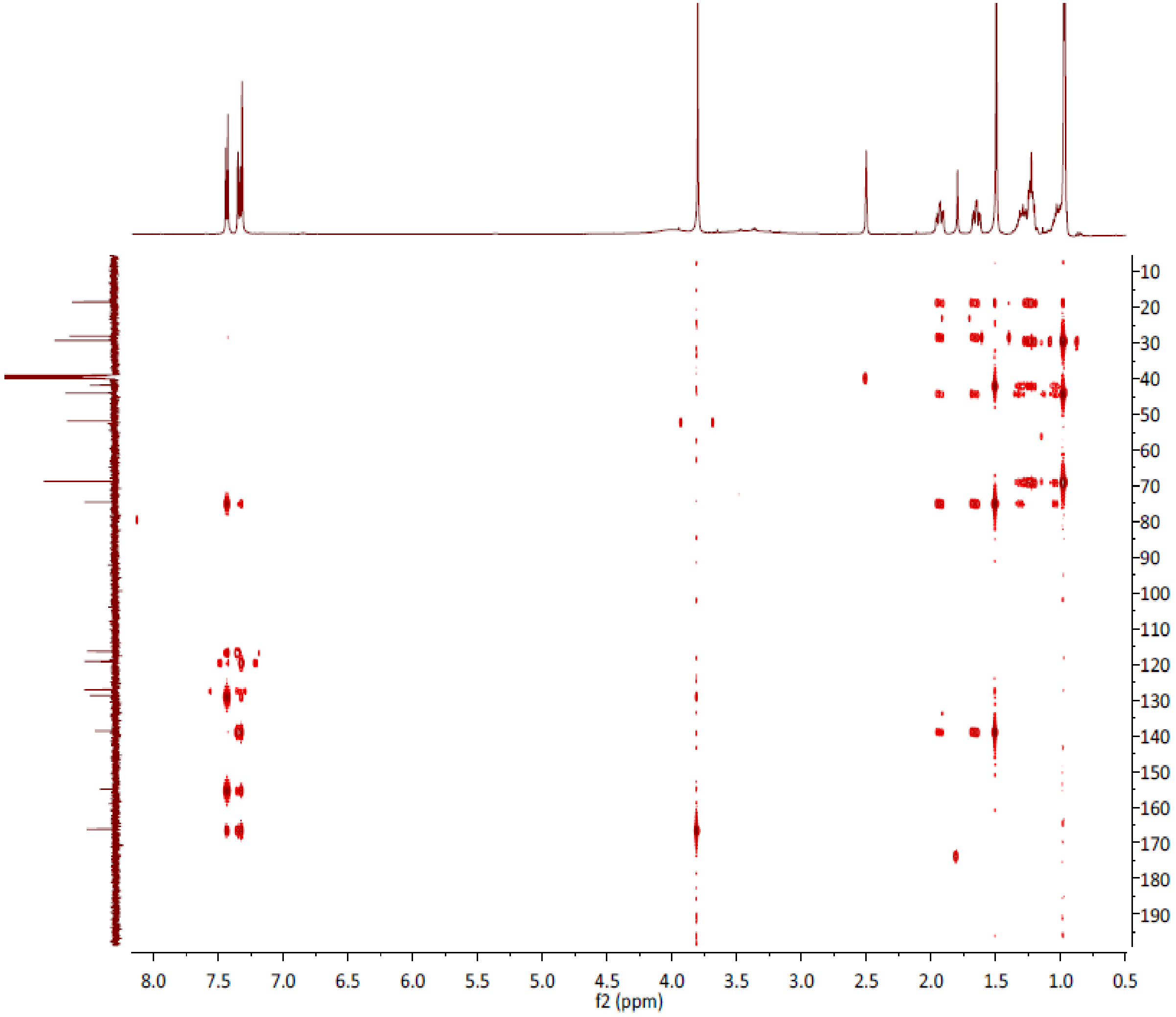

	Parameter	Value
1	Title	360$5
2	Comment	HMBC
3	Origin	Bruker BioSpin GmbH
4	Owner	nmr600
5	Spectrometer	spect
6	Solvent	DMSO
7	Temperature	298.0
8	Pulse Sequence	hmbcgplpndqf
9	Number of Scans	8
10	Receiver Gain	181
11	Relaxation Delay	1.5000
12	Pulse Width	7.9000
13	Acquisition Time	0.3760
14	Acquisition Date	2018-11-30T15:54:33
15	Modification Date	2018-11-30T16:51:36
16	G to G'	1.00000000
17	Echo Delay	0.0000
18	Gradient Duration	0.0017
19	Spectrometer Frequency	600.13
20	Spectral Width	5446.6
21	Lowest Frequency	-61.7
22	Nucleus	1H
23	Acquired Size	2048
24	Spectral Size	4096

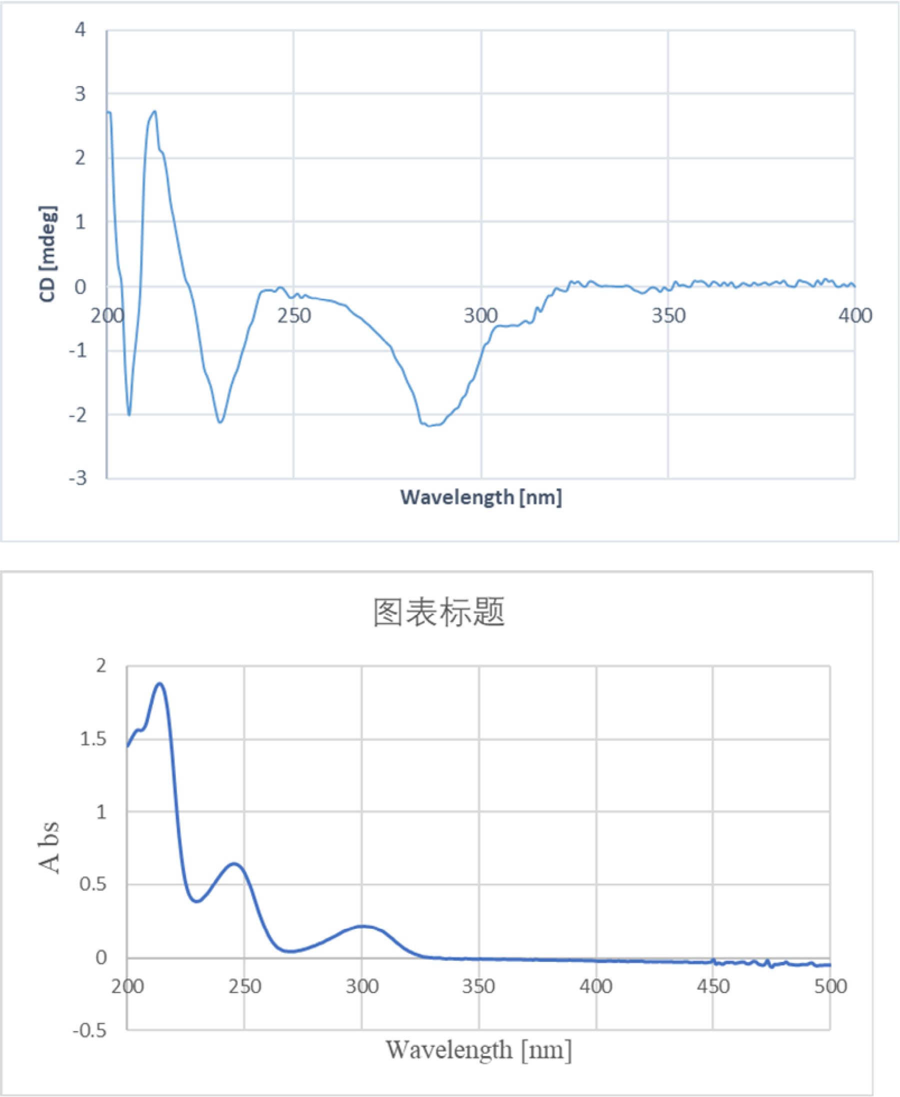

Figure S7. ECD and UV spectra of compound **1**.

Figure S8. HRESI mass spectrum of compound **2**.

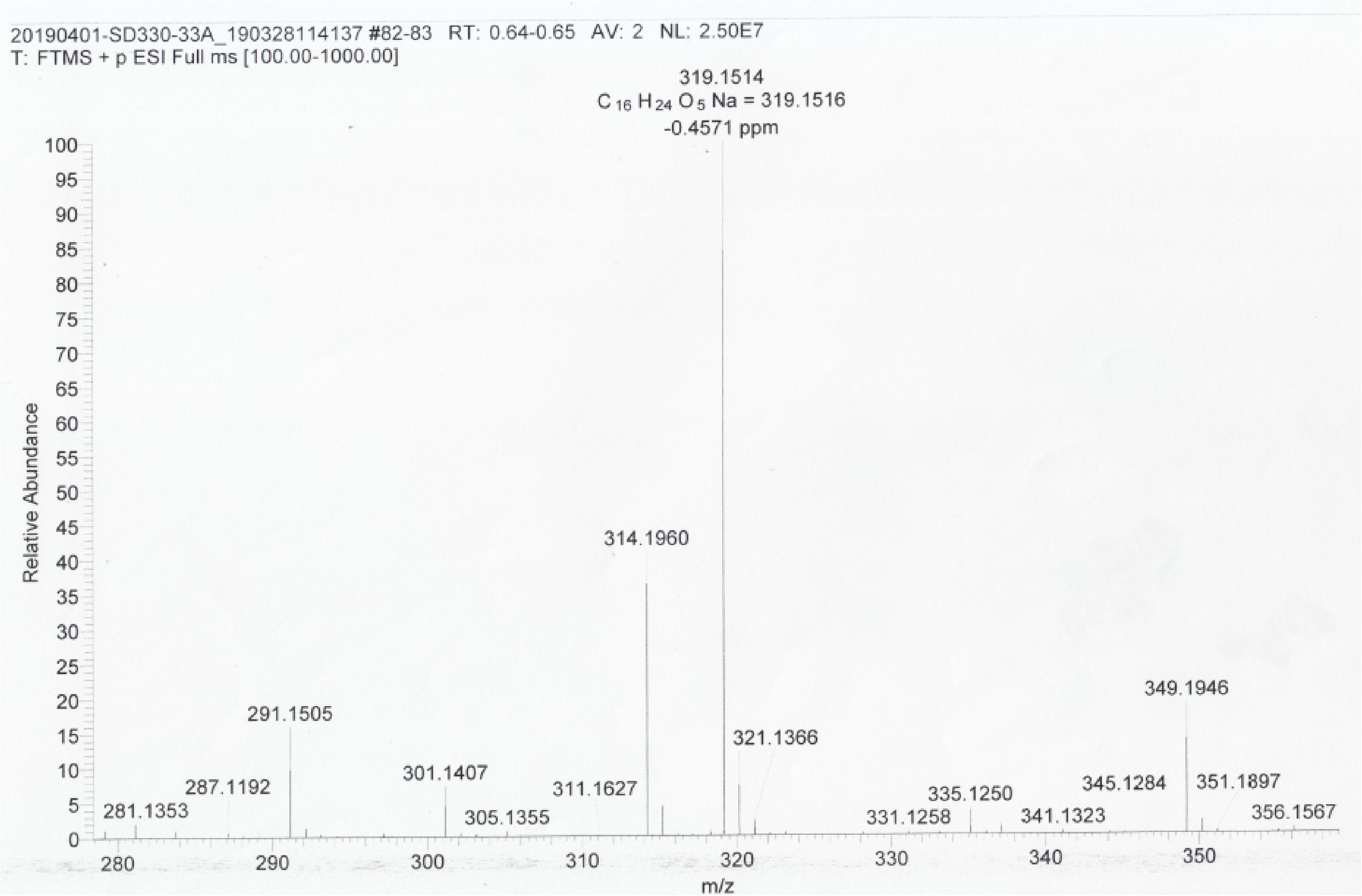

Figure S9. ^{1}H NMR (500 MHz, DMSO-d_6) spectrum of compound **2**.

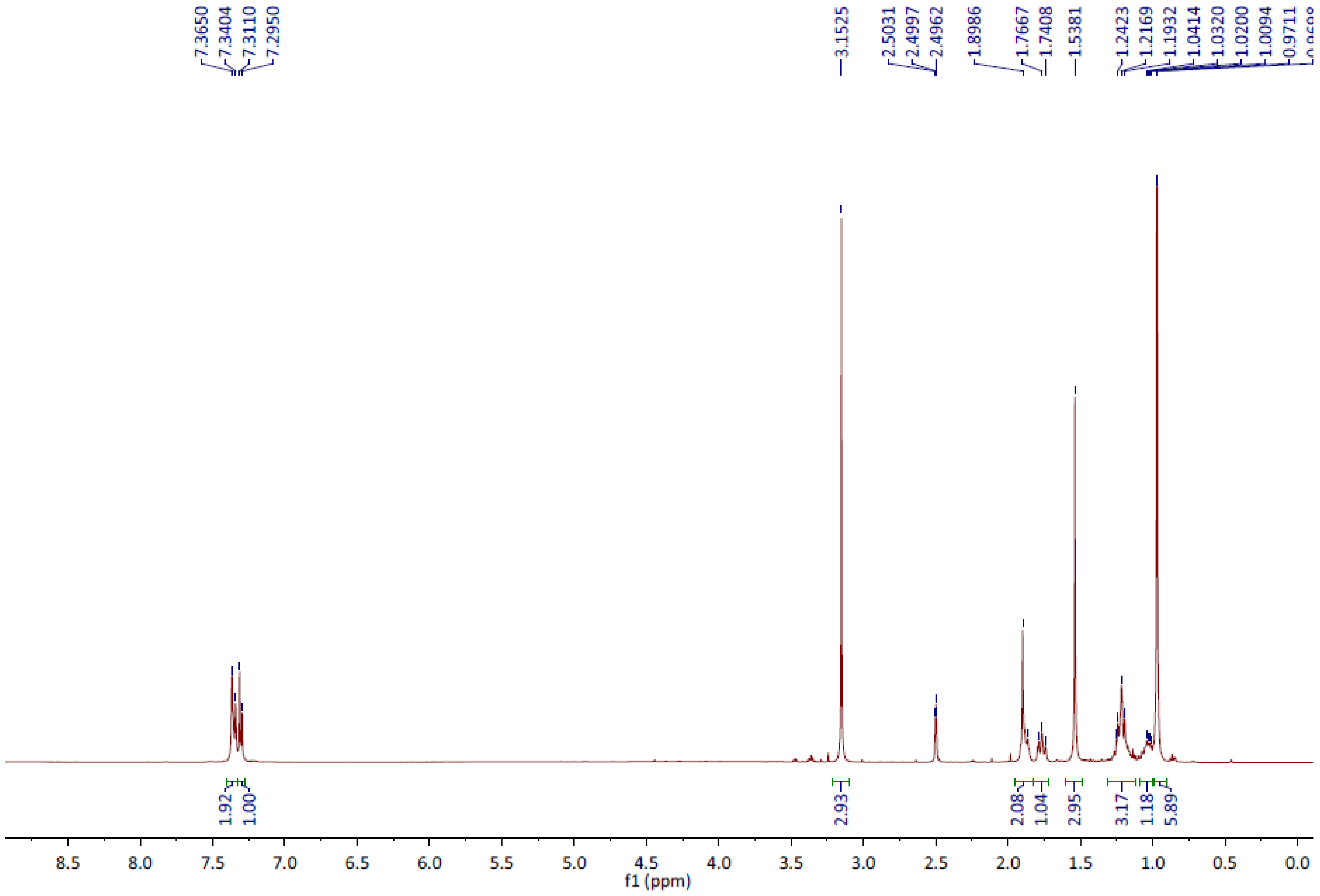

Figure S10. ^{13}C NMR (125 MHz, DMSO-d_6) and DEPT spectra of compound **2**.

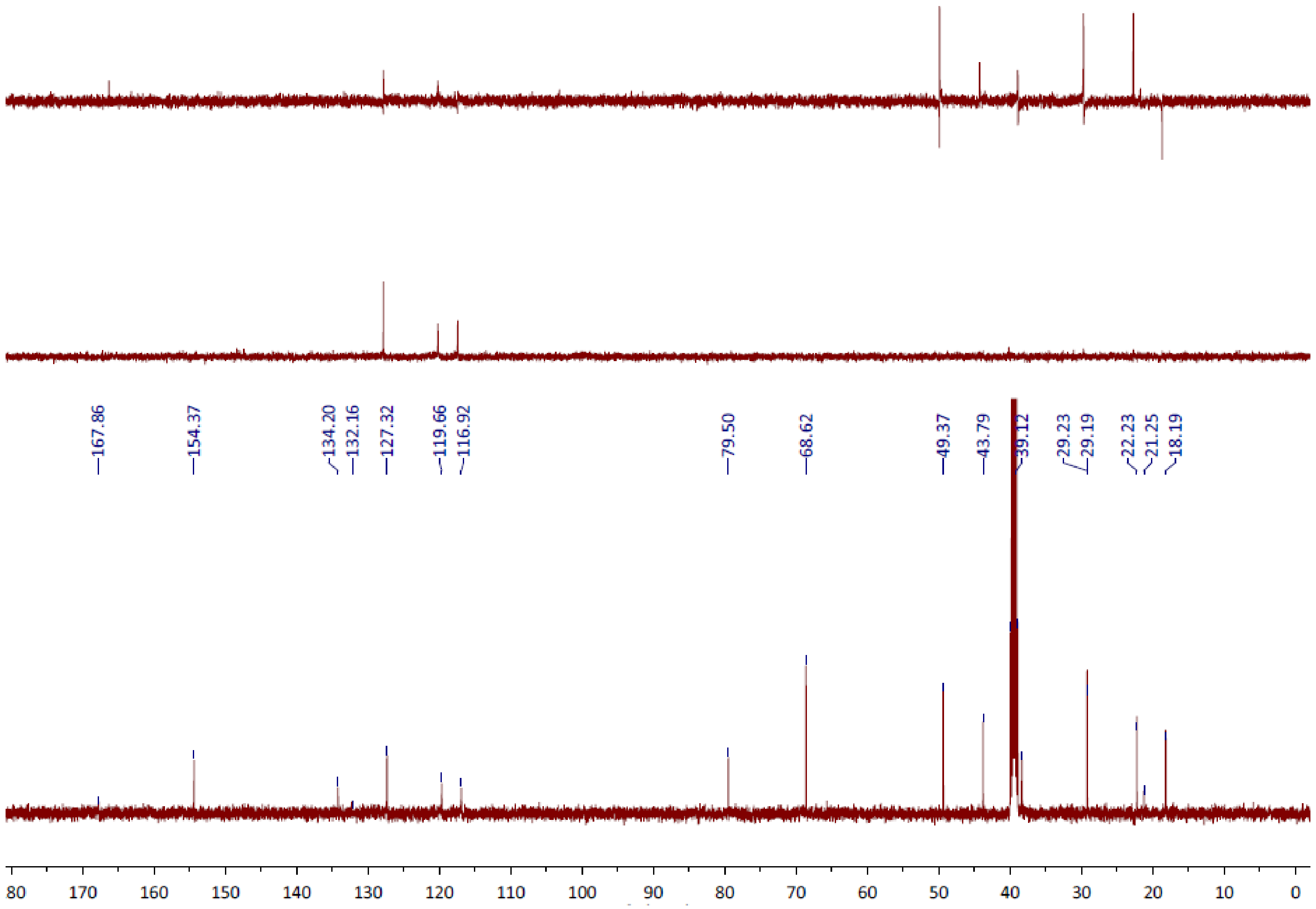

Figure S11. COSY (DMSO-d_6) spectrum of compound **2**.

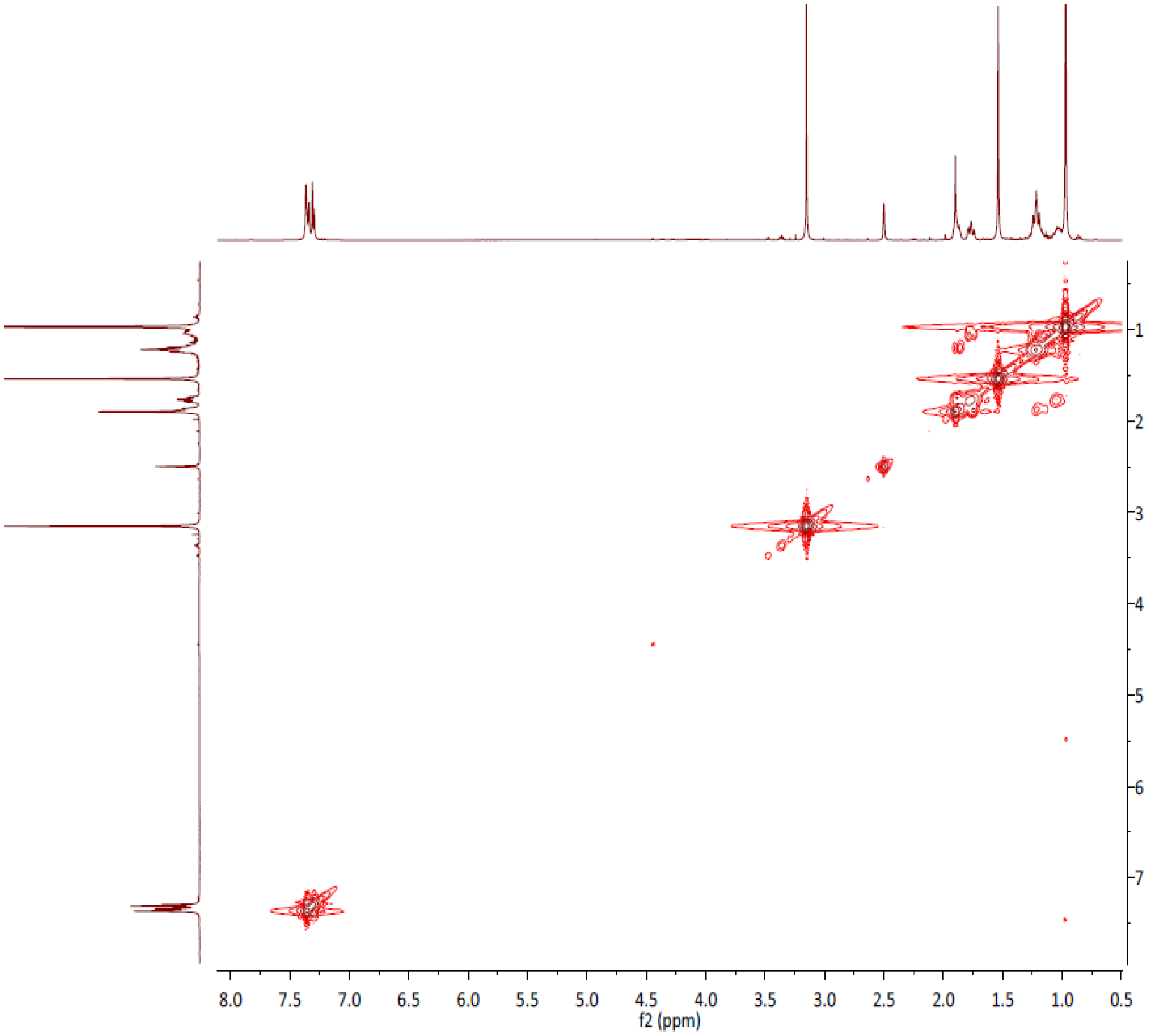

Parameters		
	Parameter	Value (f2, f1)
1	Owner	psm
2	Spectrometer	av500
3	Solvent	CDCl3
4	Temperature	298.1
5	Pulse Sequence	cosygpqf
6	Number of Scans	4
7	Receiver Gain	64
8	Relaxation Delay	1.4869
9	Pulse Width	11.7000
10	Acquisition Time	0.0935
11	Acquisition Date	2018-12-27T18:17:40
12	Modification Date	2018-12-27T18:40:52
13	G to G'	1.00000000
14	Echo Delay	0.0000
15	Gradient Duration	0.0000
16	Spectrometer Frequency	(499.80, 499.80)
17	Spectral Width	(5482.5, 5497.9)
18	Lowest Frequency	(-247.7, -255.4)
19	Nucleus	(1H, 1H)
20	Acquired Size	(512, 217)
21	Spectral Size	(512, 512)

Figure S12. HSQC (DMSO-d_6) spectrum of compound **2**.

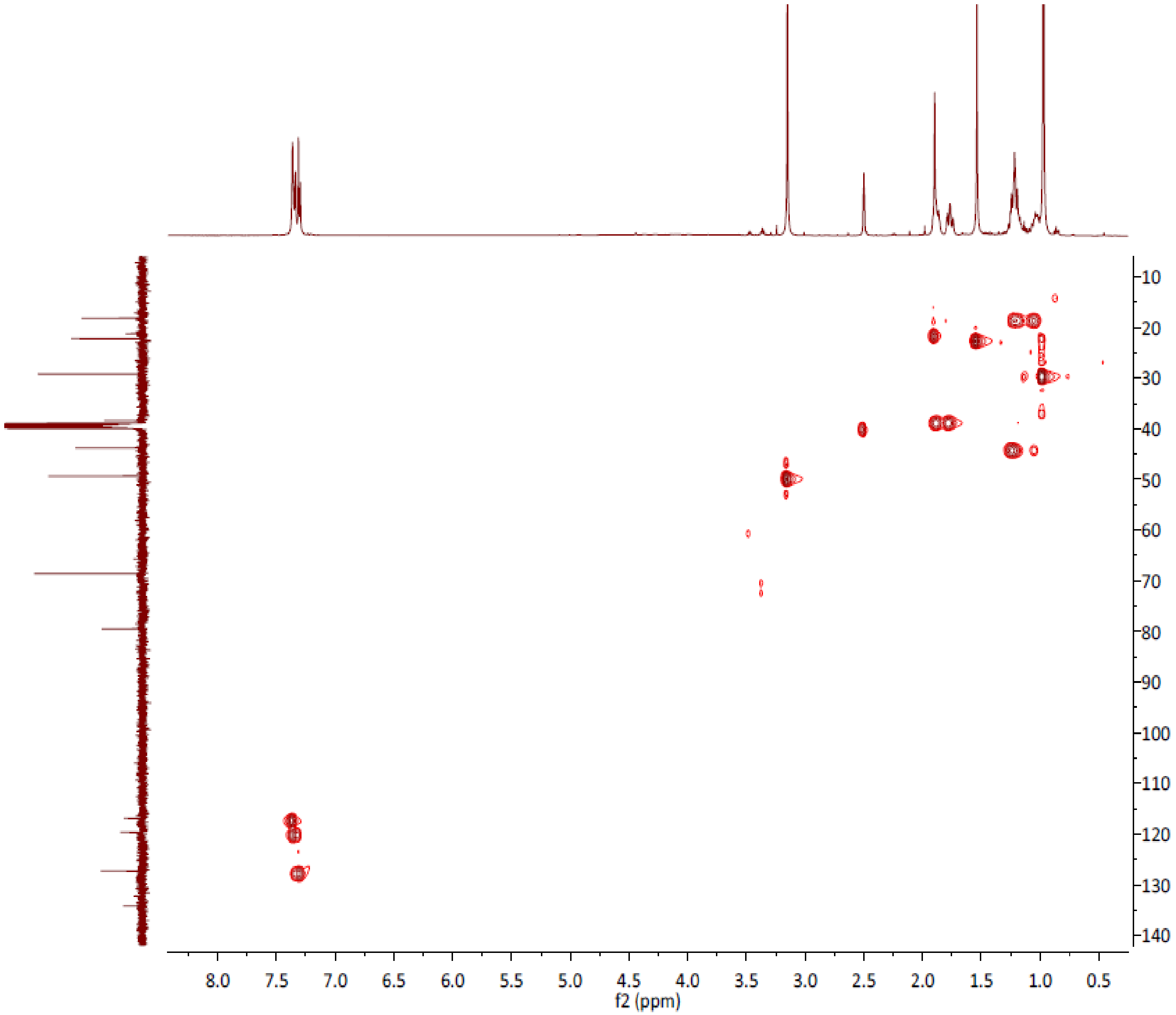

Parameters		
	Parameter	Value (f2, f1)
1	Owner	nmr600
2	Spectrometer	spect
3	Solvent	DMSO
4	Temperature	298.1
5	Pulse Sequence	hsqcetgpsisp2.2
6	Number of Scans	2
7	Receiver Gain	181
8	Relaxation Delay	1.5000
9	Pulse Width	8.1000
10	Acquisition Time	0.0784
11	Acquisition Date	2018-11-28T10:48:29
12	Modification Date	2018-11-28T11:00:15
13	G to G'	1.00000000
14	Echo Delay	0.0000
15	Gradient Duration	0.0017
16	Spectrometer Frequency	(600.13, 150.90)
17	Spectral Width	(6533.1, 25641.0)
18	Lowest Frequency	(-116.8, -1502.8)
19	Nucleus	(1H, 13C)
20	Acquired Size	(512, 220)
21	Spectral Size	(512, 512)

Figure S13. HMBC (DMSO-d_6) spectrum of compound **2**.

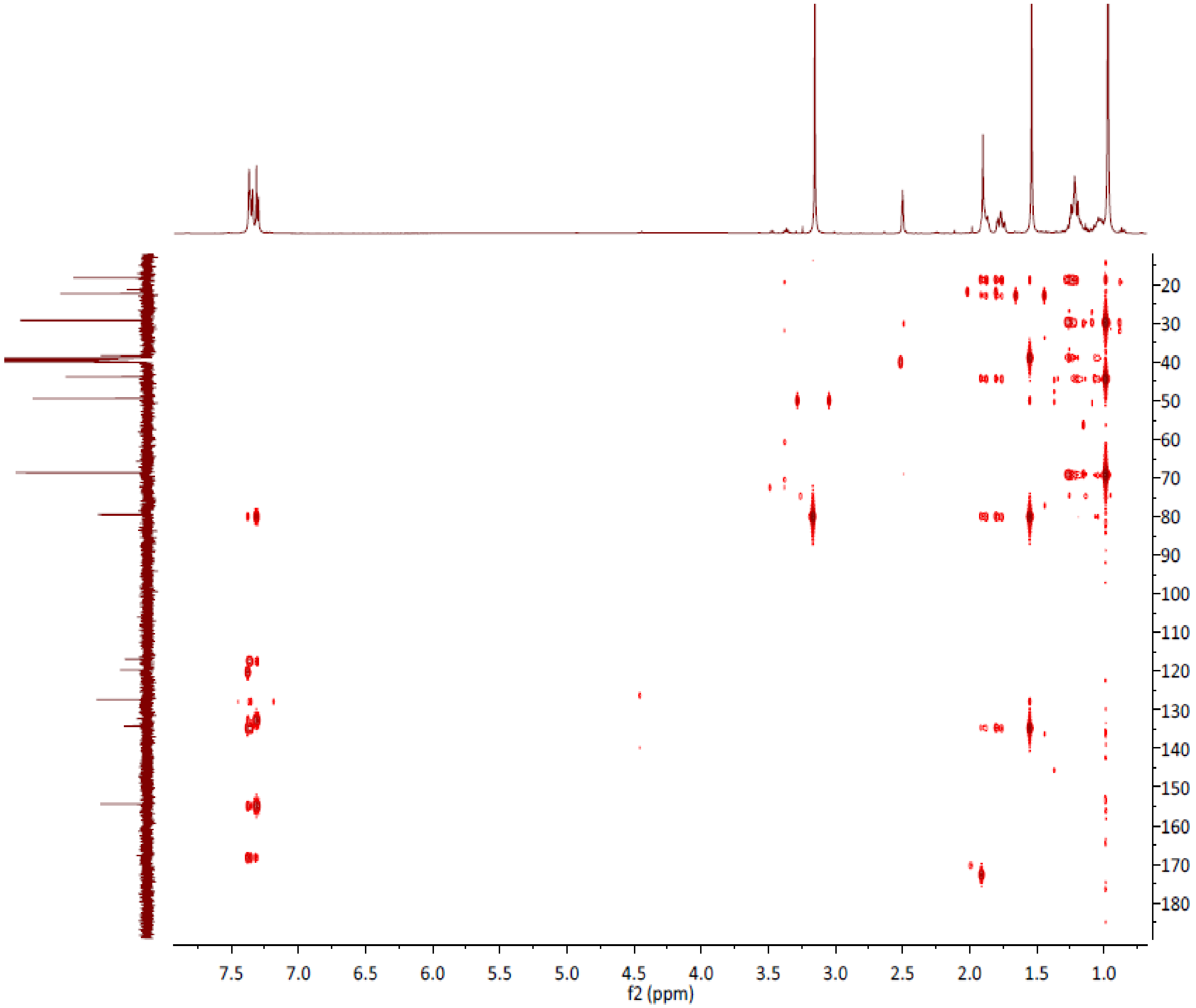

Parameters		
	Parameter	Value (f2, f1)
1	Owner	nmr600
2	Spectrometer	spect
3	Solvent	DMSO
4	Temperature	298.0
5	Pulse Sequence	hmbcgplpndqf
6	Number of Scans	8
7	Receiver Gain	181
8	Relaxation Delay	1.5000
9	Pulse Width	8.1000
10	Acquisition Time	0.3135
11	Acquisition Date	2018-11-27T15:42:47
12	Modification Date	2018-11-27T16:38:01
13	G to G'	1.00000000
14	Echo Delay	0.0000
15	Gradient Duration	0.0017
16	Spectrometer Frequency	(600.13, 150.90)
17	Spectral Width	(6533.1, 33112.6)
18	Lowest Frequency	(-116.8, -711.5)
19	Nucleus	(1H, 13C)
20	Acquired Size	(2048, 220)
21	Spectral Size	(2048, 1024)

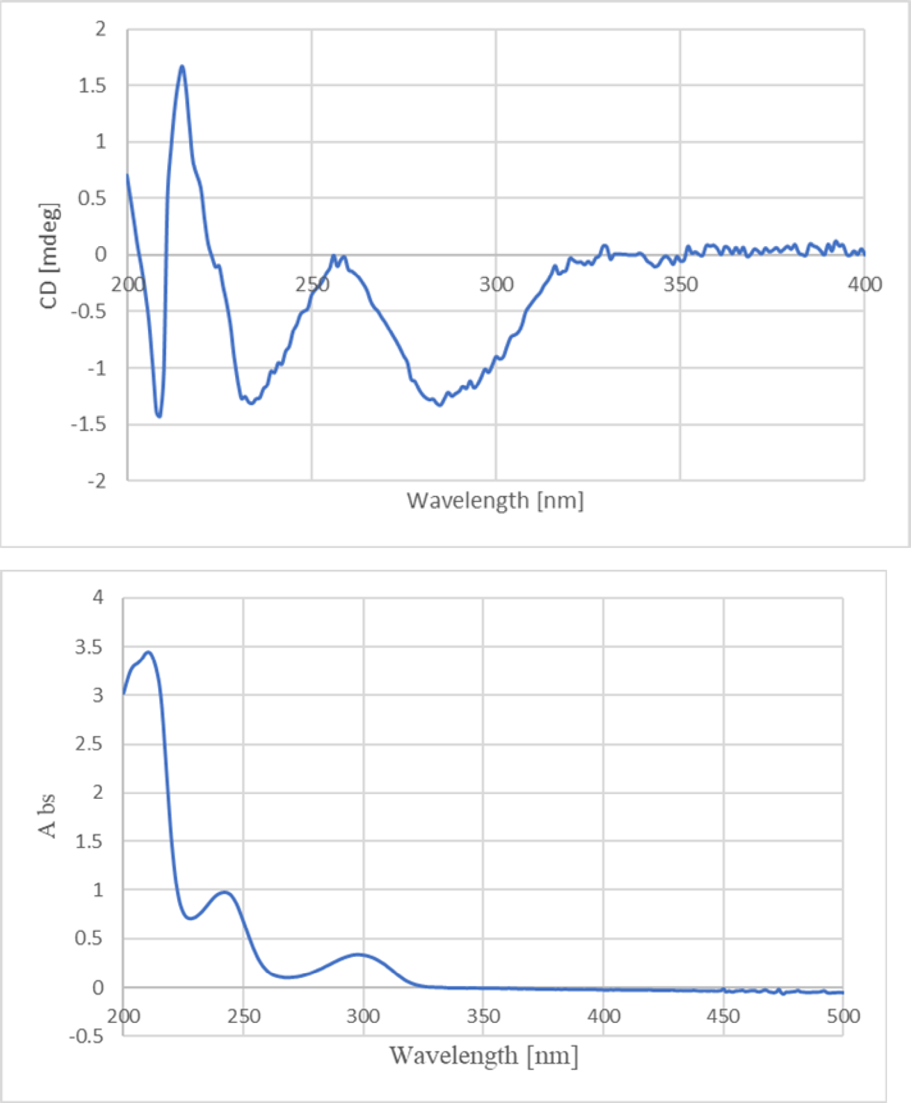

Figure S14. ECD and UV spectra of compound **2**.

Figure S15. HPLC profile of the crude fermented extract.

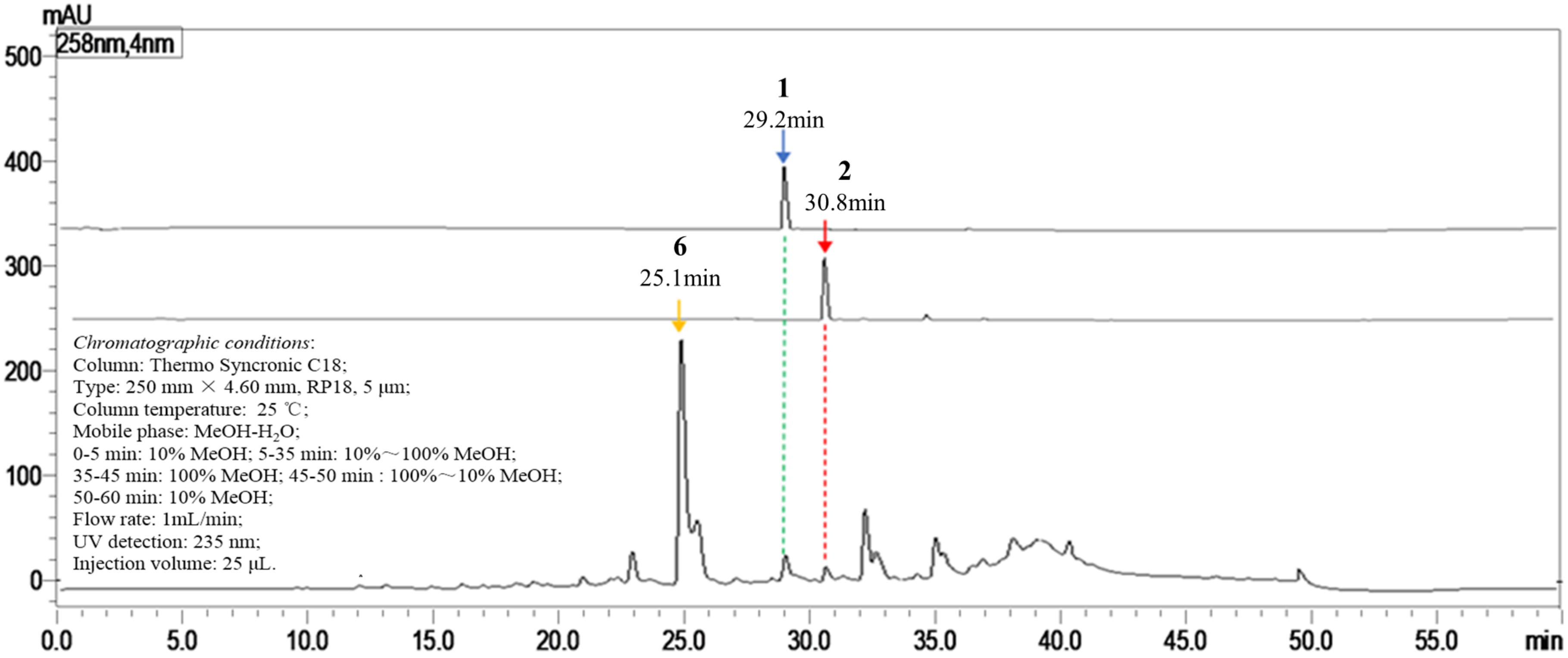

Figure S16. HRESI mass spectrum of compound **3**.

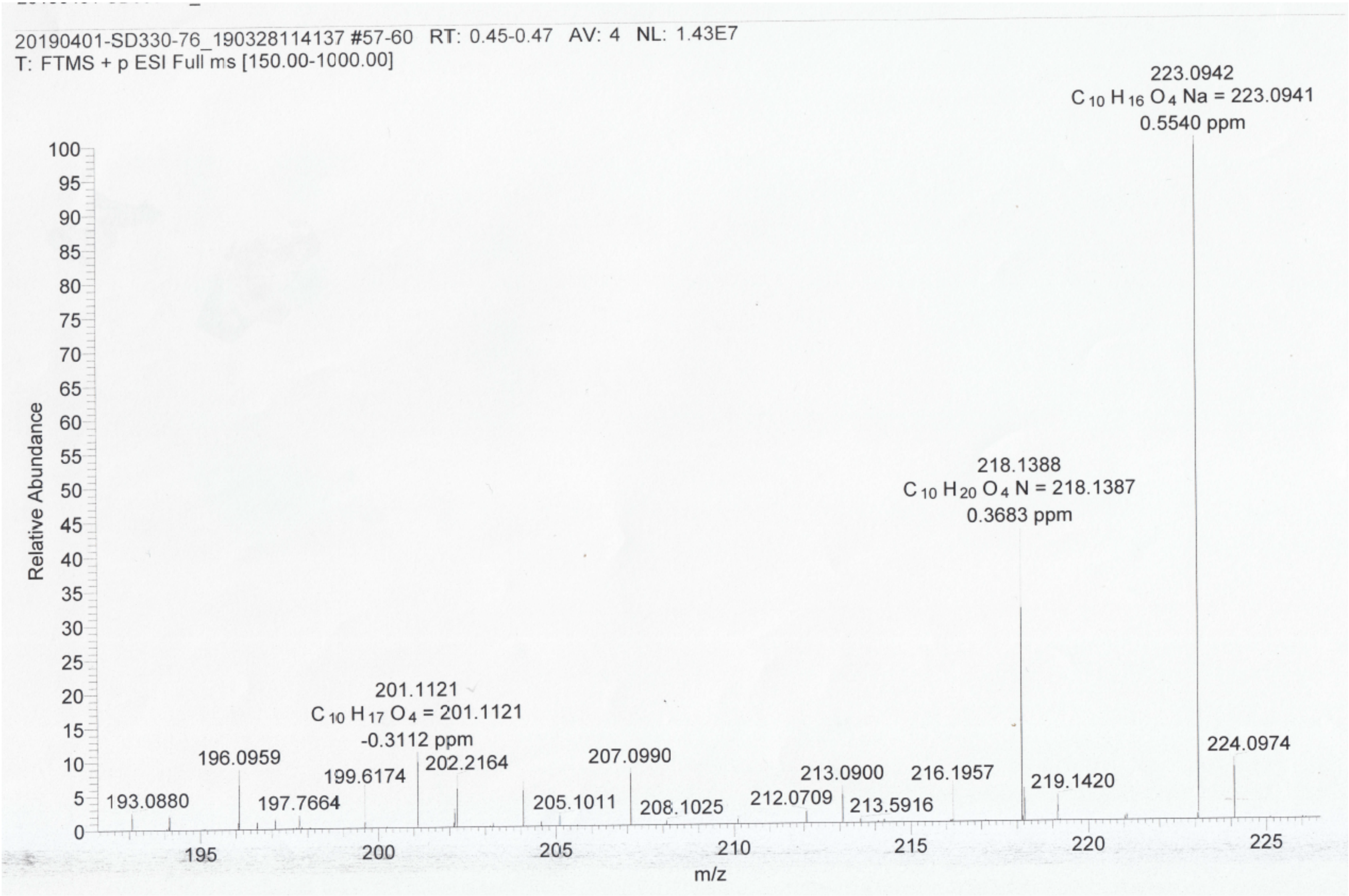

Figure S17. ^{1}H NMR (500 MHz, DMSO-d_6) spectrum of compound **3**.

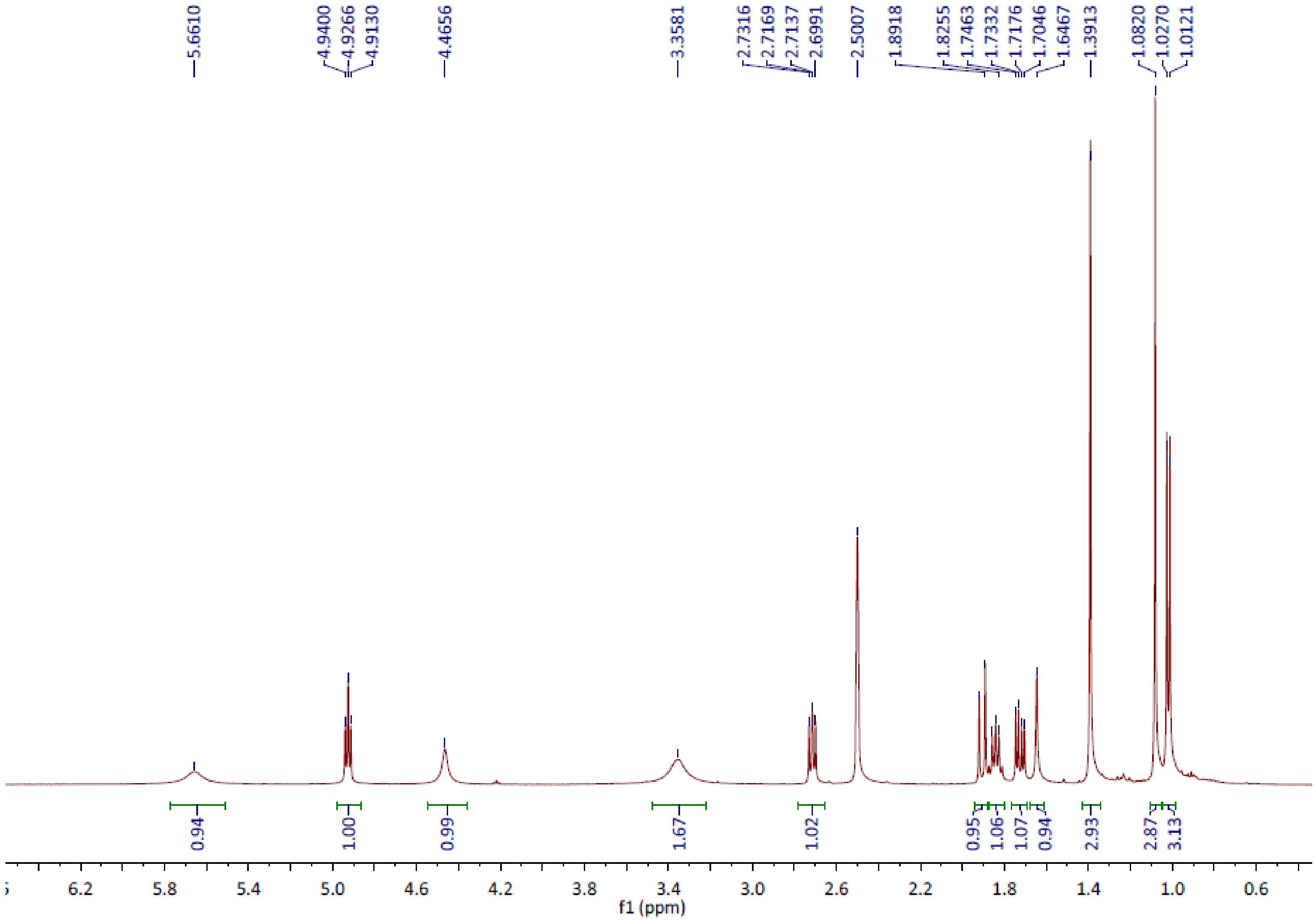

Figure S18. ^{13}C NMR (125 MHz, DMSO-d_6) and DEPT spectra of compound **3**.

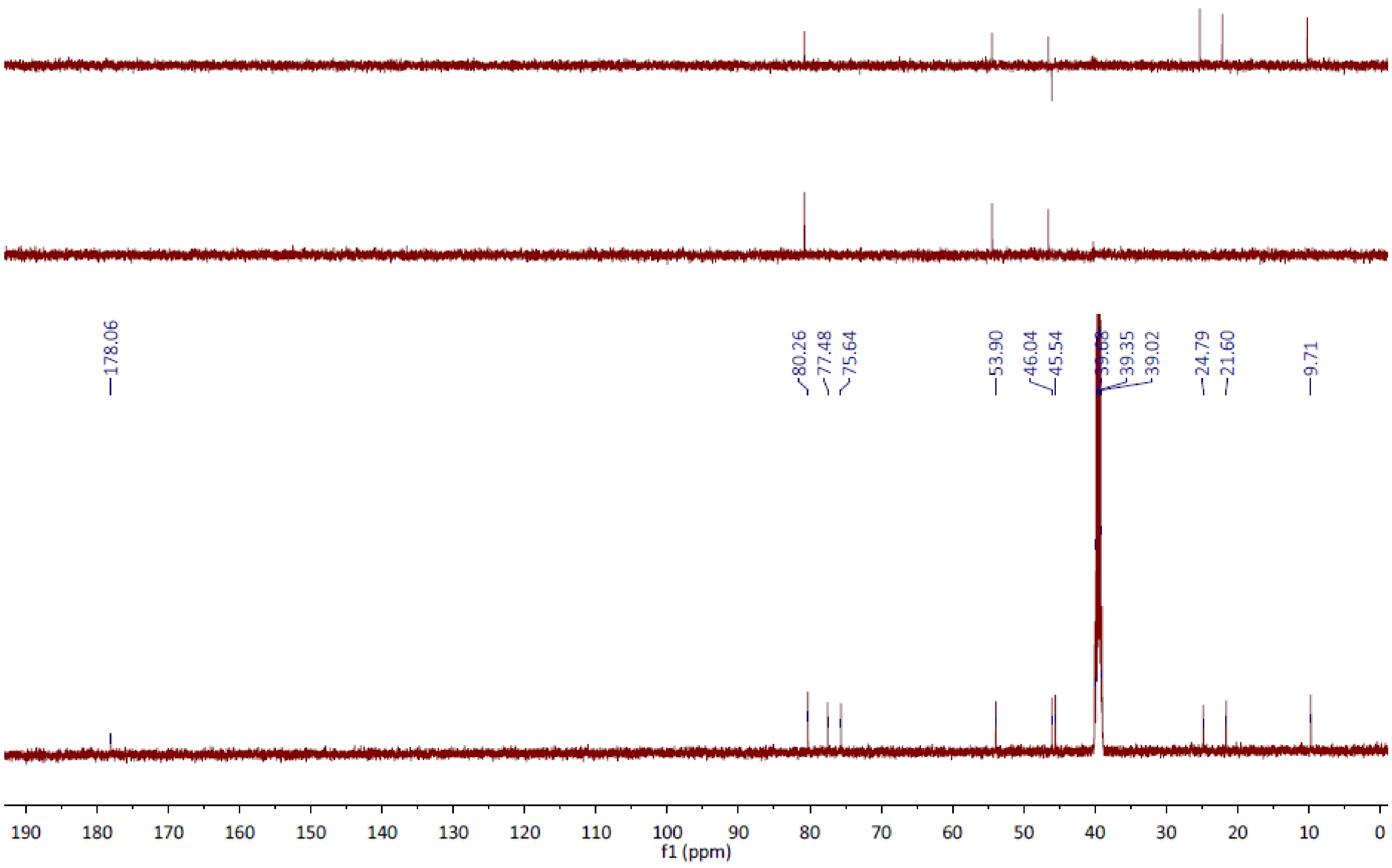

Figure S19. COSY (DMSO-d_6) spectrum of compound **3**.

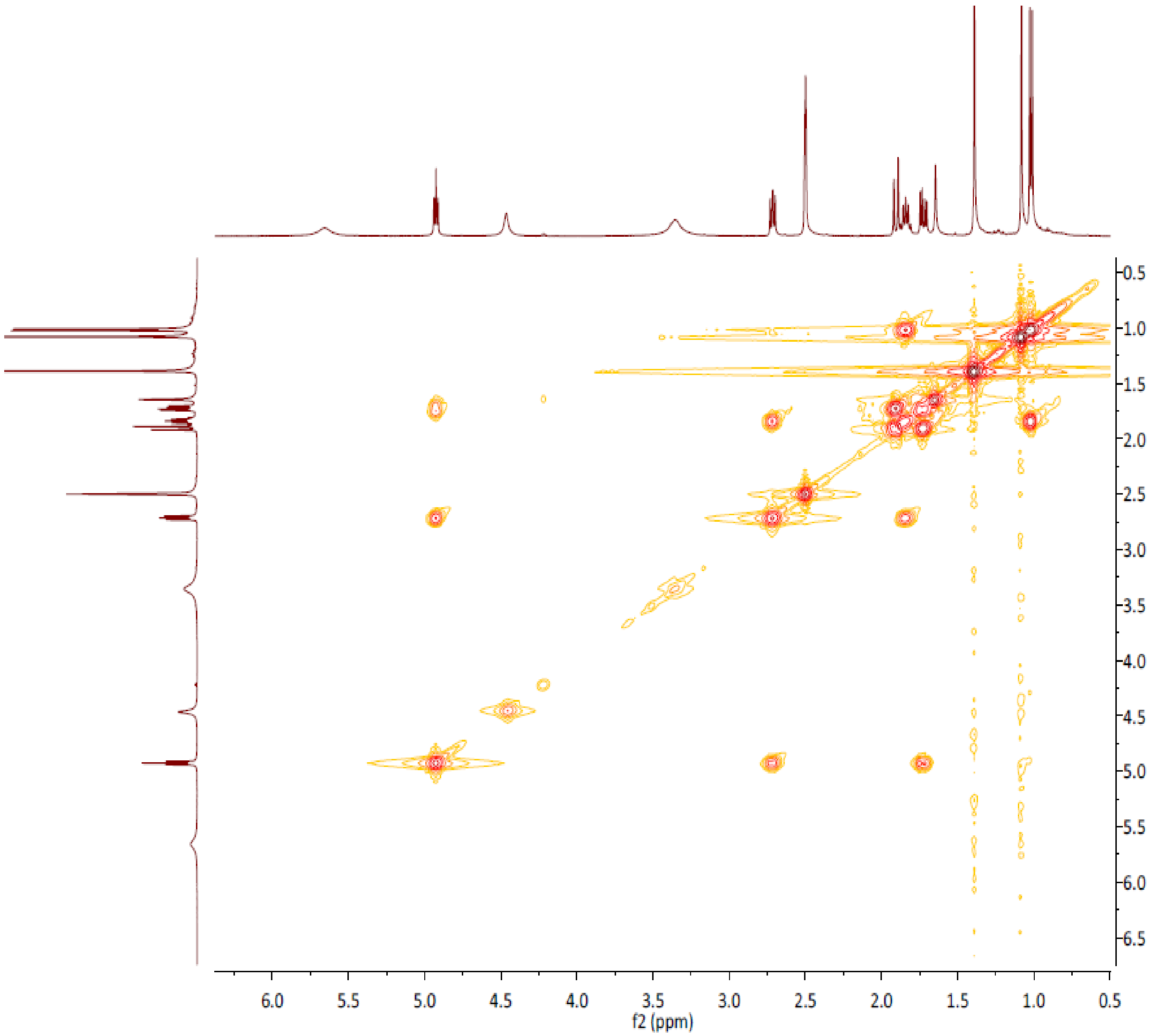

Parameters		
	Parameter	Value (f2, f1)
1	Owner	psm
2	Spectrometer	av500
3	Temperature	298.6
4	Pulse Sequence	cosygpqf
5	Number of Scans	4
6	Receiver Gain	64
7	Relaxation Delay	1.4869
8	Pulse Width	11.7000
9	Acquisition Time	0.0934
10	Acquisition Date	2019-01-08T14:58:27
11	Modification Date	2019-01-08T15:25:53
12	G to G'	1.00000000
13	Echo Delay	0.0000
14	Gradient Duration	0.0000
15	Spectrometer Frequency	(499.80, 499.80)
16	Spectral Width	(5482.5, 5497.9)
17	Lowest Frequency	(-247.2, -254.9)
18	Nucleus	(1H, 1H)
19	Acquired Size	(512, 256)
20	Spectral Size	(512, 512)

Figure S20. HSQC (DMSO-d_6) spectrum of compound **3**.

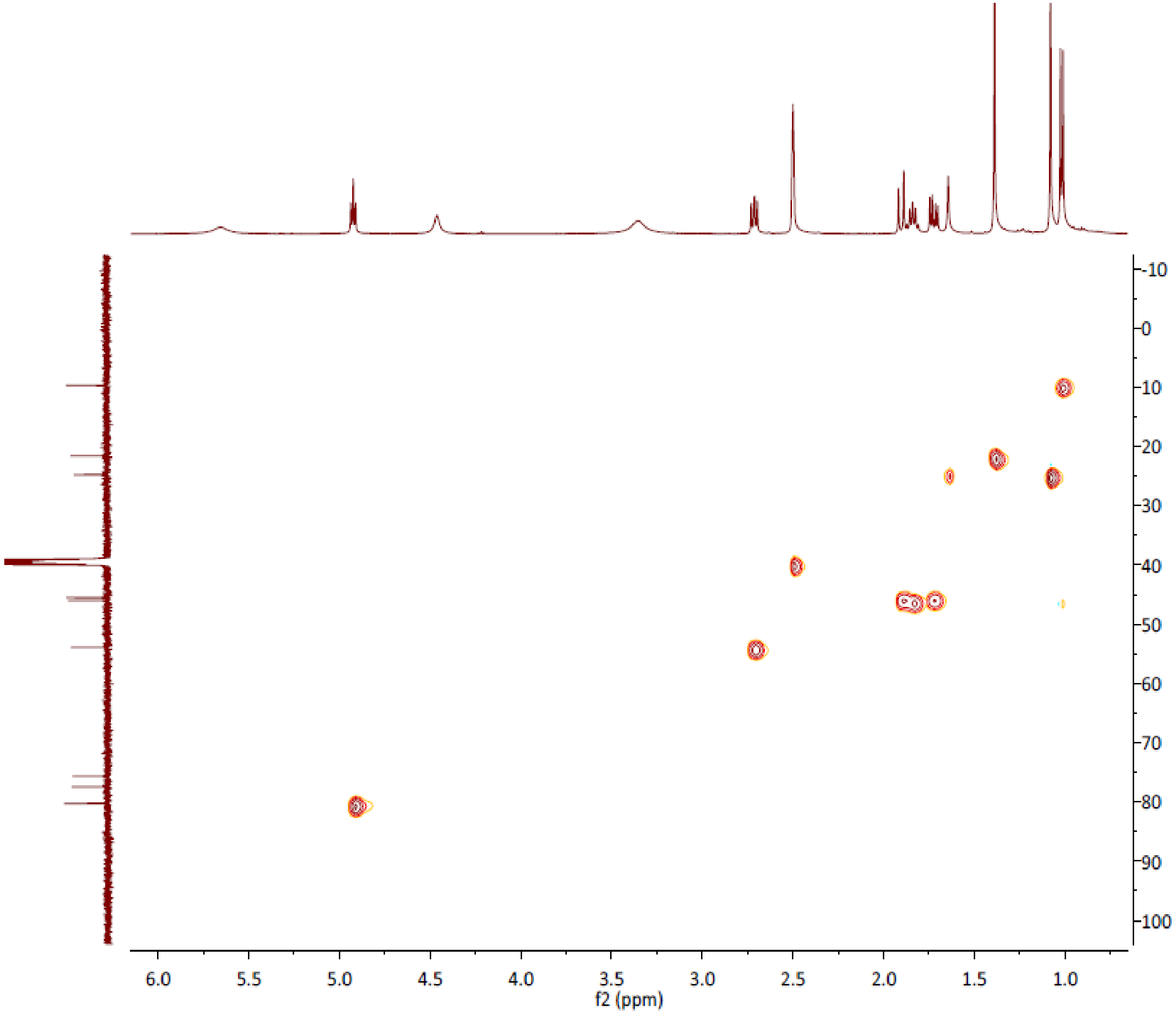

Parameters		
	Parameter	Value (f2, f1)
1	Owner	psm
2	Spectrometer	av500
3	Temperature	298.8
4	Pulse Sequence	hsqcetgpsi2
5	Number of Scans	8
6	Receiver Gain	18390
7	Relaxation Delay	1.5000
8	Pulse Width	11.7000
9	Acquisition Time	0.0640
10	Acquisition Date	2019-01-08T15:26:47
11	Modification Date	2019-01-08T16:21:43
12	G to G'	1.00000000
13	Echo Delay	0.0000
14	Gradient Duration	0.0000
15	Spectrometer Frequency	(499.80, 125.67)
16	Spectral Width	(5482.5, 31421.8)
17	Lowest Frequency	(-253.6, -4401.1)
18	Nucleus	(1H, 13C)
19	Acquired Size	(512, 256)
20	Spectral Size	(512, 512)

Figure S21. HMBC (DMSO-d_6) spectrum of compound **3**.

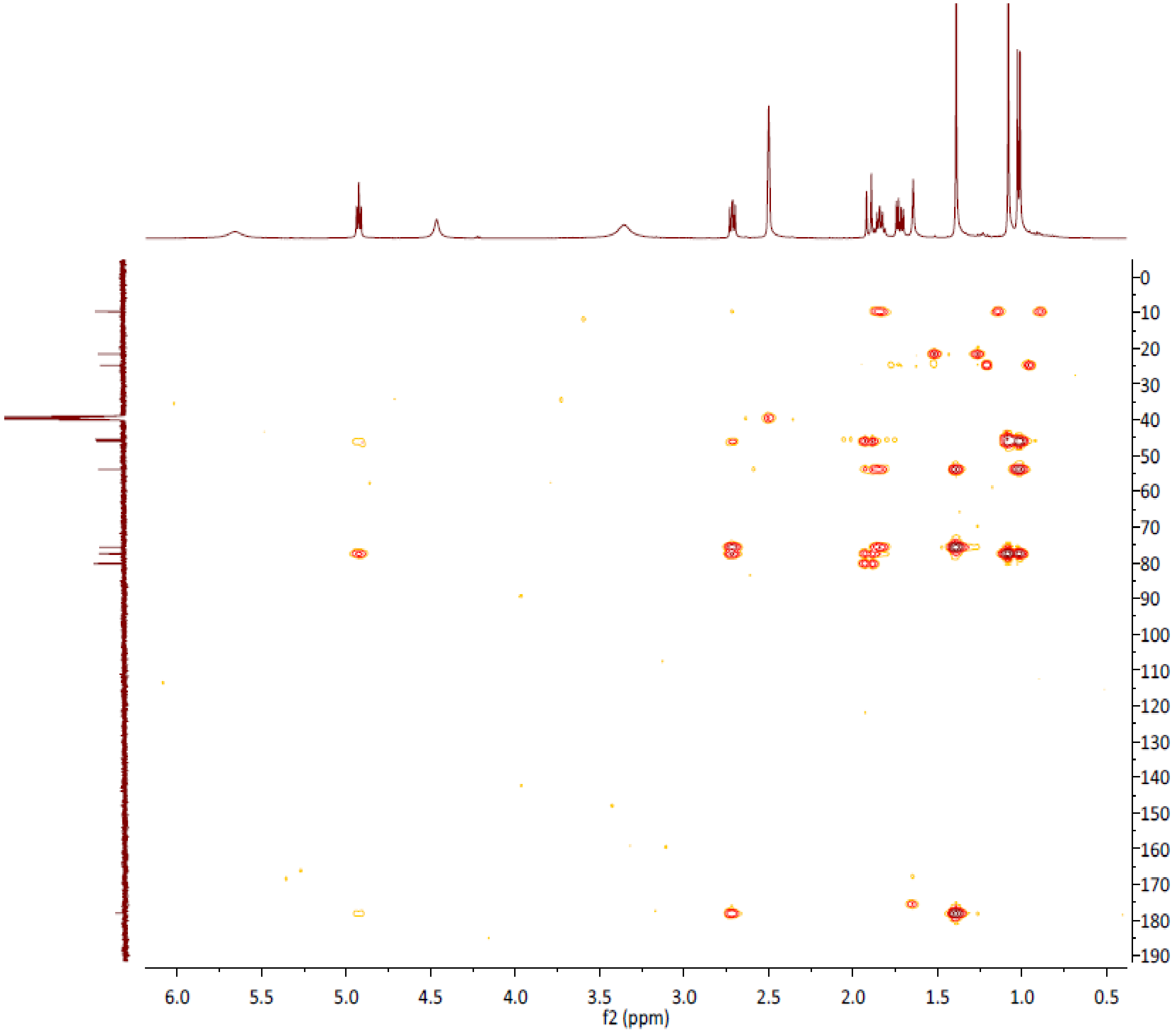

Parameters		
	Parameter	Value (f2, f1)
1	Owner	psm
2	Spectrometer	av500
3	Solvent	DMSO
4	Temperature	299.0
5	Pulse Sequence	hmbcgplpndqf
6	Number of Scans	8
7	Receiver Gain	16384
8	Relaxation Delay	1.5000
9	Pulse Width	11.7000
10	Acquisition Time	0.1574
11	Acquisition Date	2019-01-08T16:22:35
12	Modification Date	2019-01-08T17:19:01
13	G to G'	1.00000000
14	Echo Delay	0.0000
15	Gradient Duration	0.0000
16	Spectrometer Frequency	(499.80, 125.67)
17	Spectral Width	(5482.5, 32679.7)
18	Lowest Frequency	(-247.2, -1955.8)
19	Nucleus	(1H, 13C)
20	Acquired Size	(512, 256)
21	Spectral Size	(512, 512)

Figure S22. NOESY (DMSO-d_6) spectrum of compound **3**.

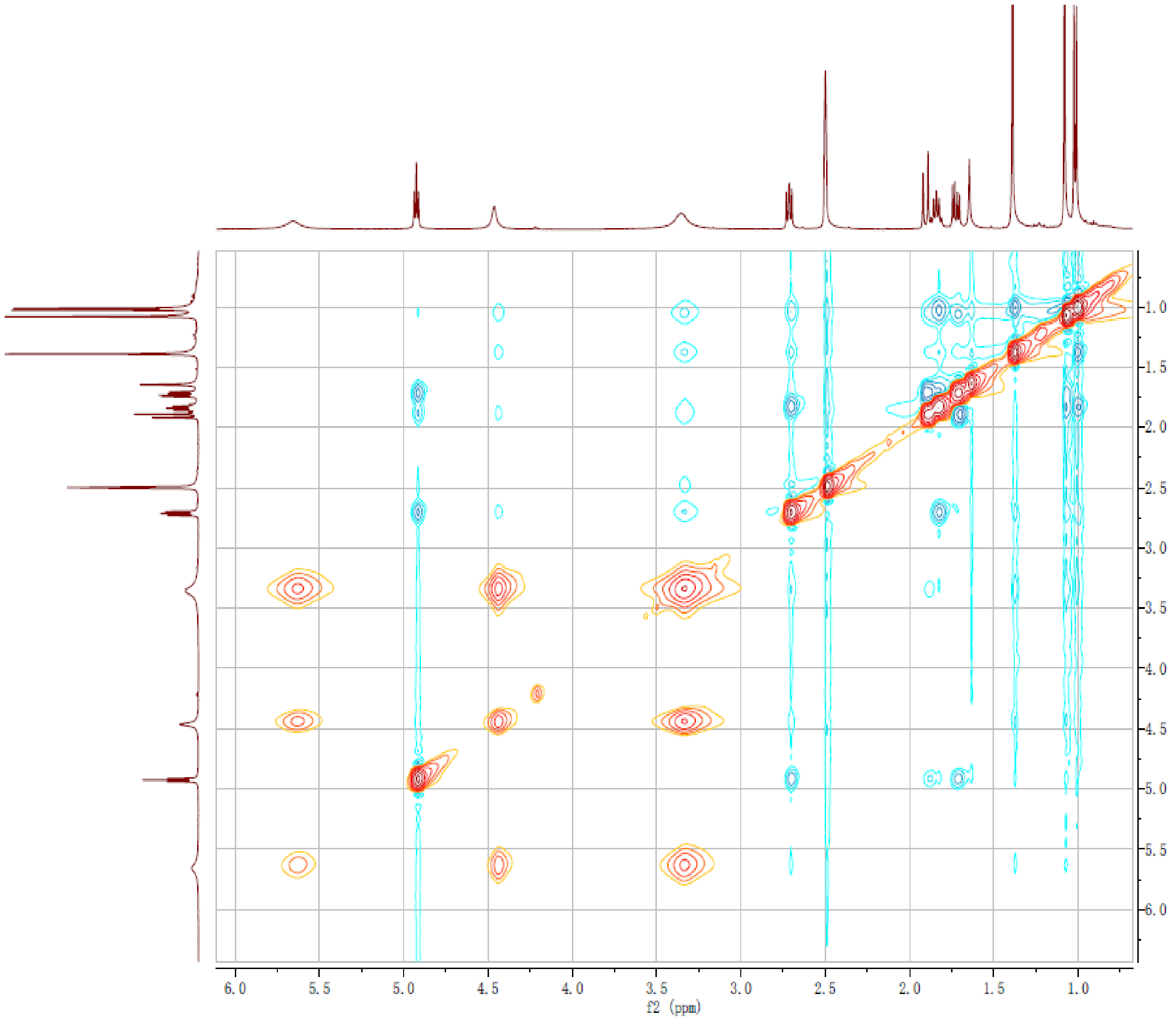

Parameters		
	Parameter	Value (f2, f1)
1	Owner	root
2	Spectrometer	av500
3	Temperature	298.4
4	Pulse Sequence	noesyph
5	Number of Scans	16
6	Receiver Gain	64
7	Relaxation Delay	2.0000
8	Pulse Width	11.7000
9	Acquisition Time	0.0934
10	Acquisition Date	2019-01-08T17:20:24
11	Modification Date	2019-01-08T20:10:21
12	G to G'	1.00000000
13	Echo Delay	0.0000
14	Gradient Duration	0.0000
15	Spectrometer Frequency	(499.80, 499.80)
16	Spectral Width	(5482.5, 5497.9)
17	Lowest Frequency	(-246.9, -254.6)
18	Nucleus	(1H, 1H)
19	Acquired Size	(512, 212)
20	Spectral Size	(512, 512)

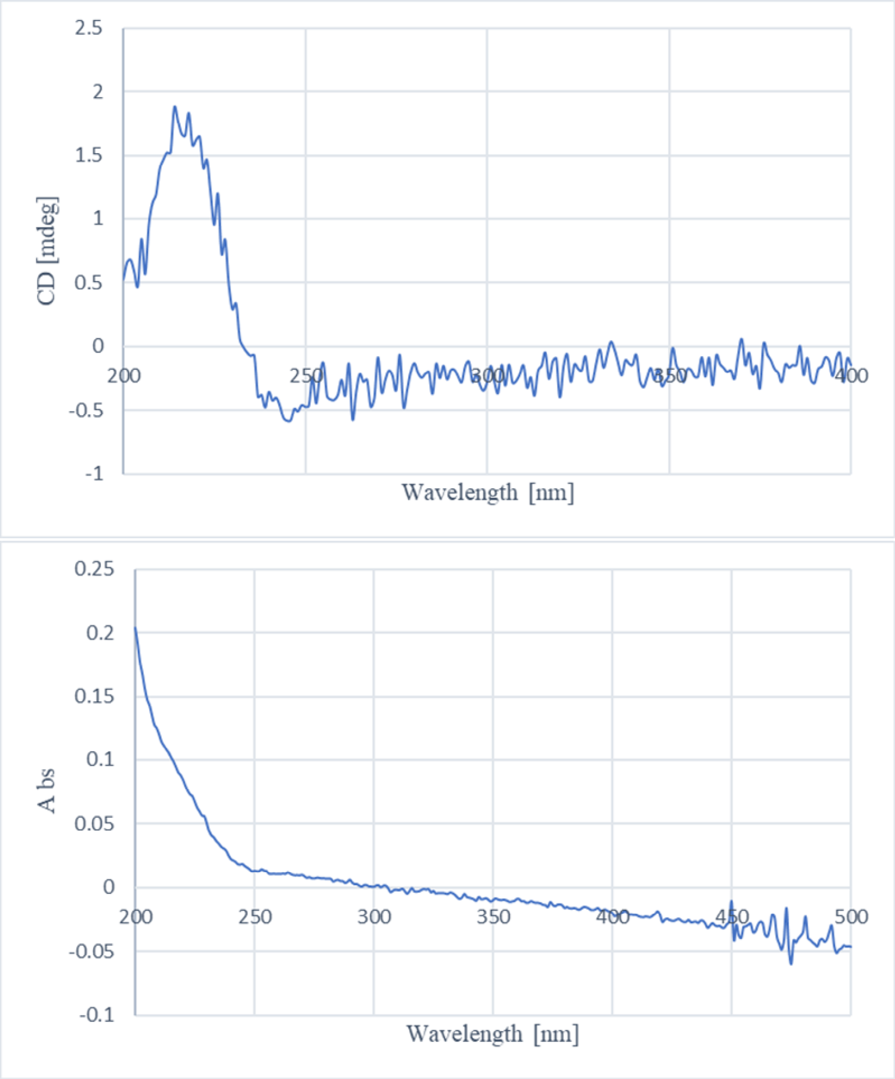

Figure S23. ECD and UV spectra of compound **3**.

Figure S24. HRESI mass spectrum of compound **4**.

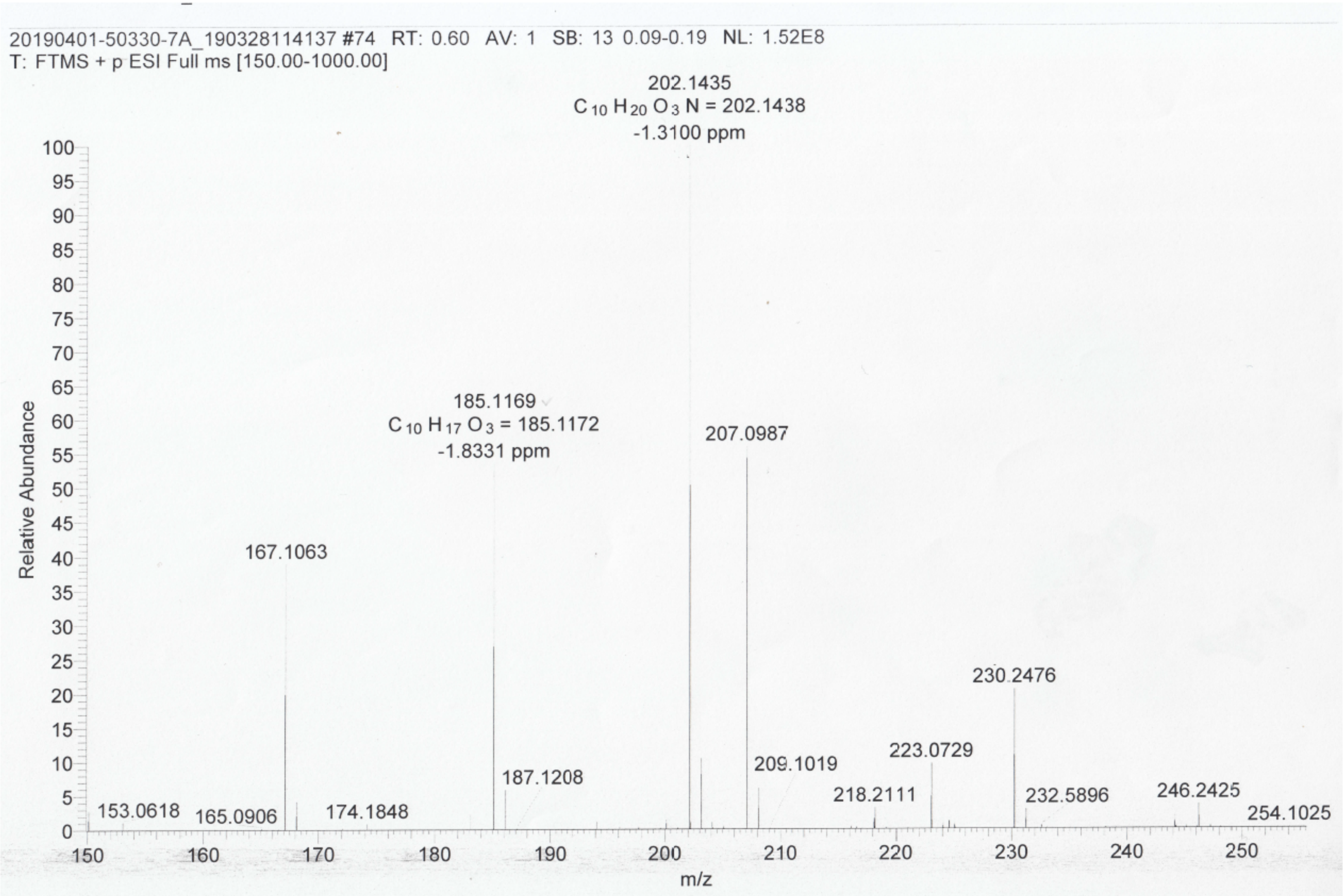

Figure S25. ^{1}H NMR (500 MHz, DMSO-d_6) spectrum of compound **4**.

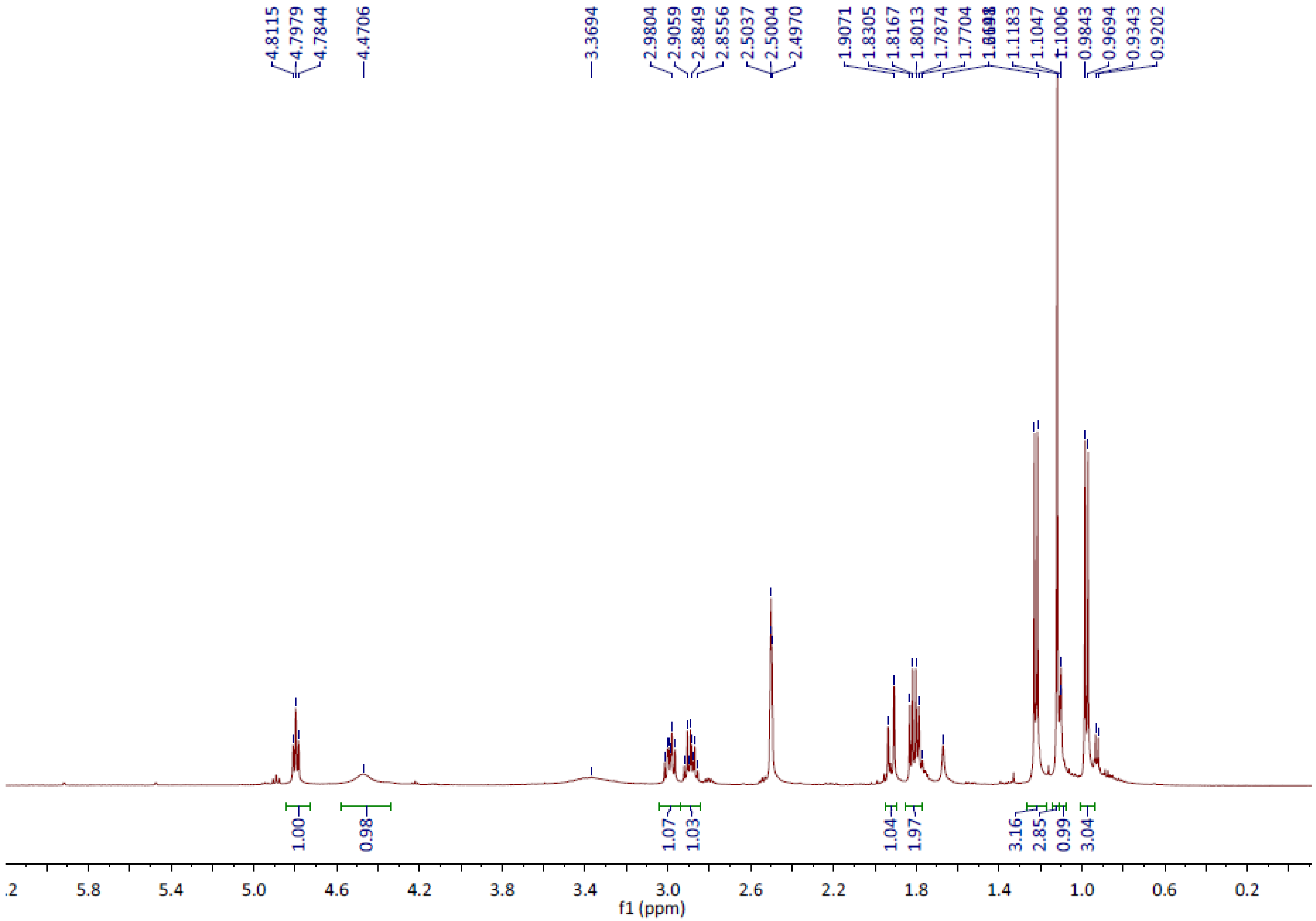

Figure S26. ^{13}C NMR (125 MHz, DMSO-d_6) and DEPT spectra of compound **4**.

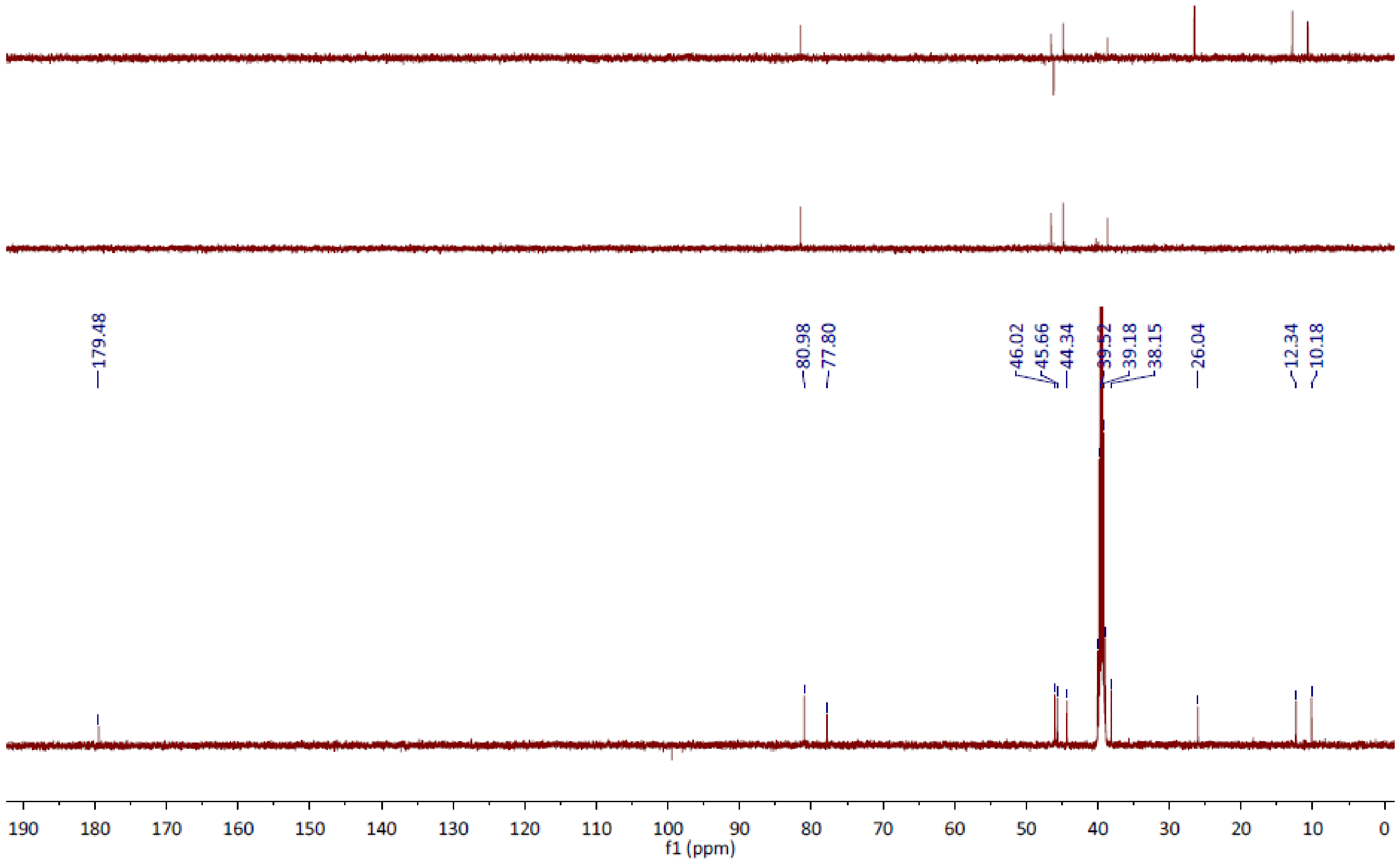

Figure S27. COSY (DMSO-d_6) spectrum of compound **4**.

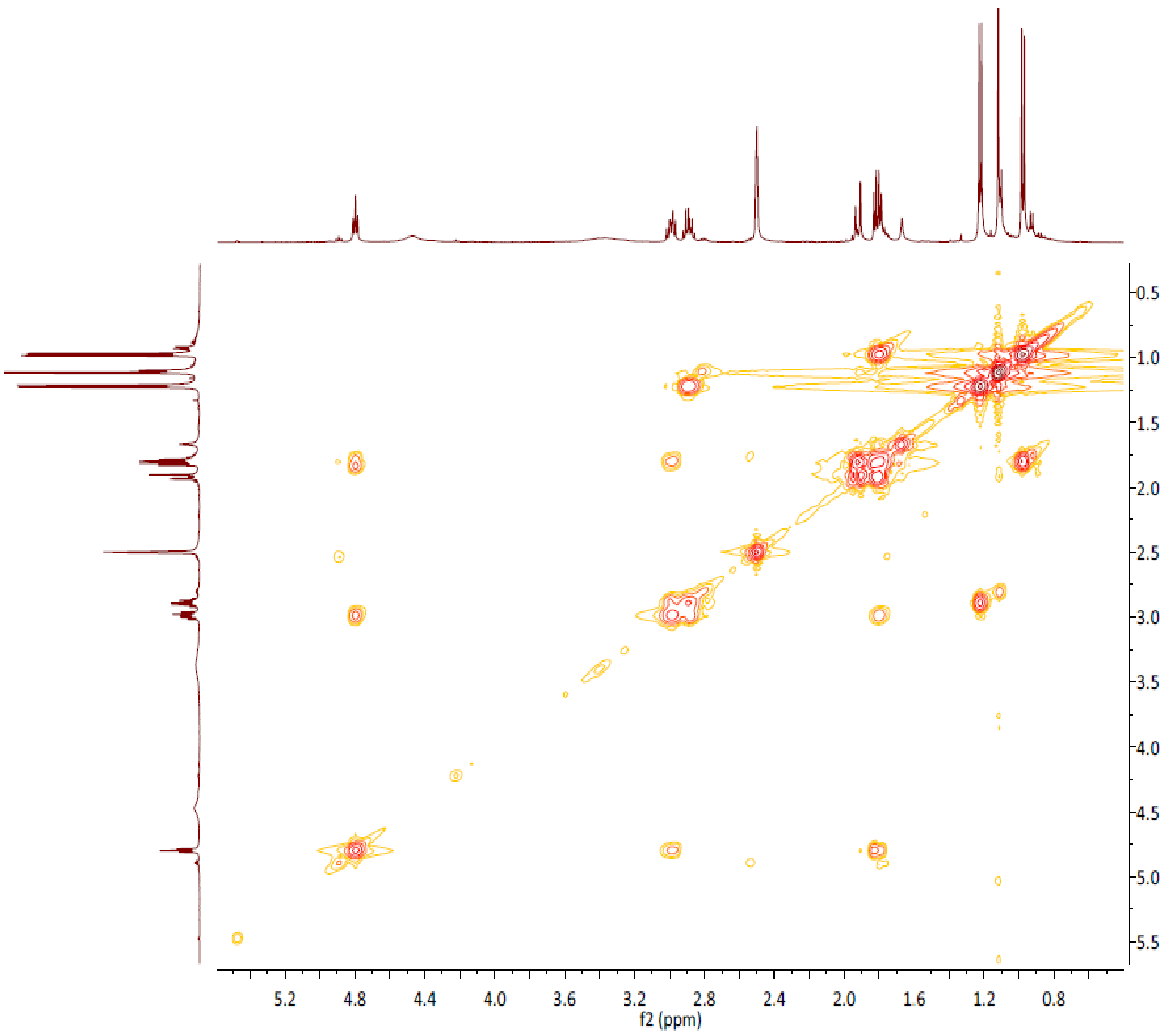

Parameters		
	Parameter	Value (f2, f1)
1	Owner	psm
2	Spectrometer	av500
3	Temperature	299.4
4	Pulse Sequence	cosygpqf
5	Number of Scans	4
6	Receiver Gain	64
7	Relaxation Delay	1.4869
8	Pulse Width	11.7000
9	Acquisition Time	0.0934
10	Acquisition Date	2019-01-07T19:48:27
11	Modification Date	2019-01-07T20:15:52
12	G to G'	1.00000000
13	Echo Delay	0.0000
14	Gradient Duration	0.0000
15	Spectrometer Frequency	(499.80, 499.80)
16	Spectral Width	(5482.5, 5497.9)
17	Lowest Frequency	(-247.2, -254.9)
18	Nucleus	(1H, 1H)
19	Acquired Size	(512, 256)
20	Spectral Size	(512, 512)

Figure S28. HSQC (DMSO-d_6) spectrum of compound **4**.

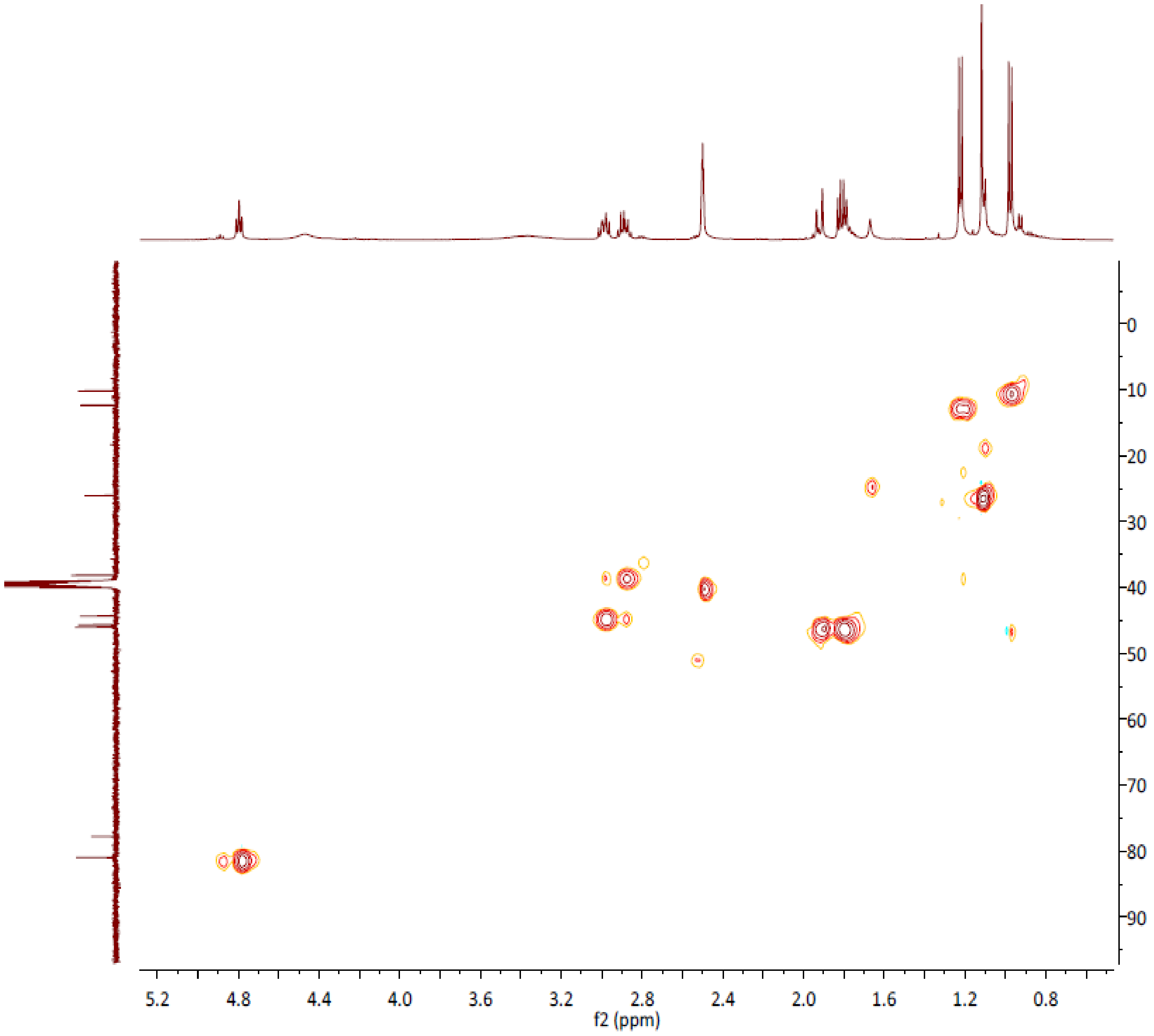

Parameters		
	Parameter	Value (f2, f1)
1	Owner	psm
2	Spectrometer	av500
3	Temperature	299.4
4	Pulse Sequence	hsqcetgpsi2
5	Number of Scans	8
6	Receiver Gain	18390
7	Relaxation Delay	1.5000
8	Pulse Width	11.7000
9	Acquisition Time	0.0640
10	Acquisition Date	2019-01-07T20:16:46
11	Modification Date	2019-01-07T21:11:42
12	G to G'	1.00000000
13	Echo Delay	0.0000
14	Gradient Duration	0.0000
15	Spectrometer Frequency	(499.80, 125.67)
16	Spectral Width	(5482.5, 31421.8)
17	Lowest Frequency	(-253.6, -4401.1)
18	Nucleus	(1H, 13C)
19	Acquired Size	(512, 256)
20	Spectral Size	(512, 512)

Figure S29. HMBC (DMSO-d_6) spectrum of compound **4**.

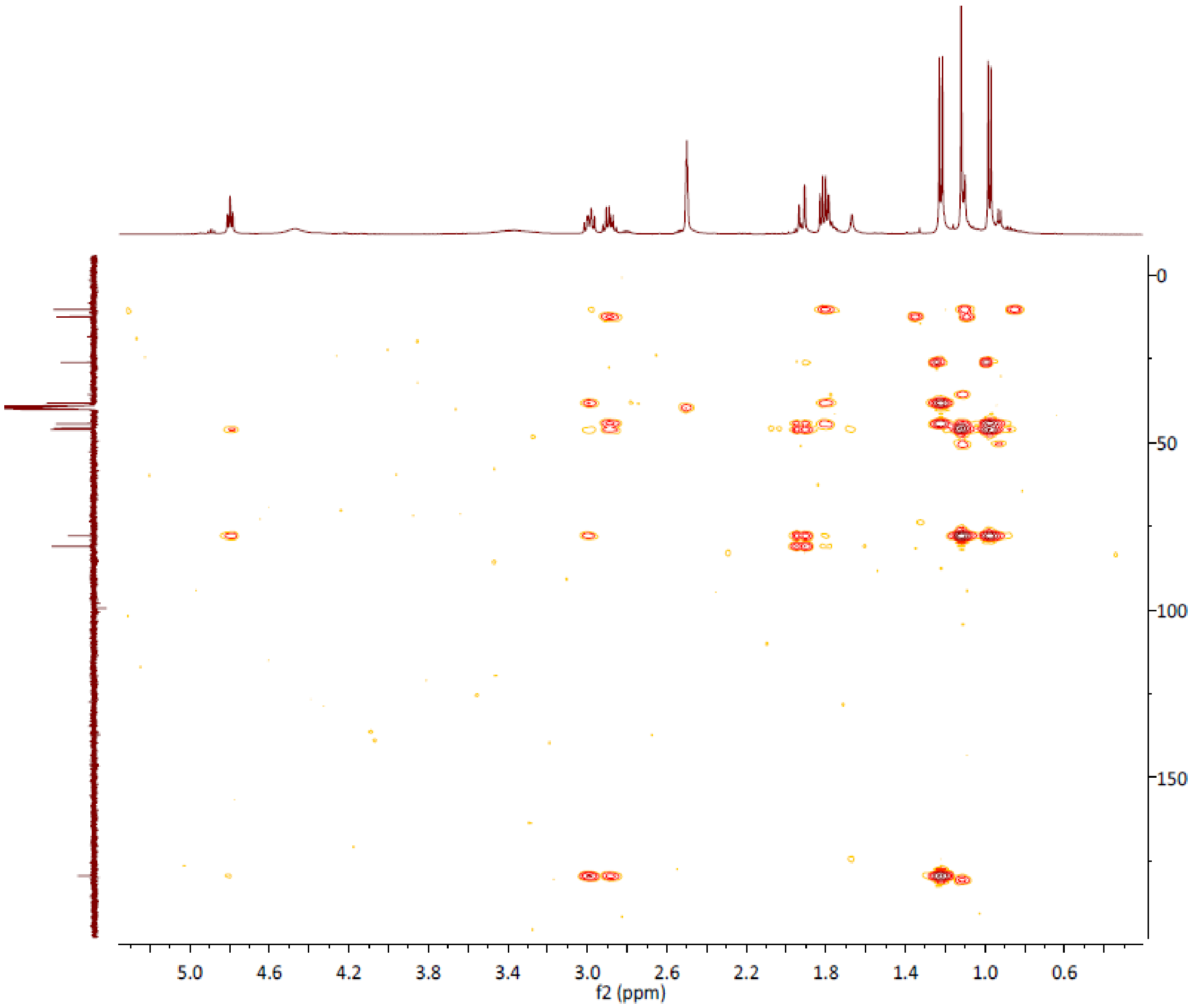

Parameters		
	Parameter	Value (f2, f1)
1	Owner	psm
2	Spectrometer	av500
3	Solvent	DMSO
4	Temperature	299.7
5	Pulse Sequence	hmbcgplpndqf
6	Number of Scans	8
7	Receiver Gain	16384
8	Relaxation Delay	1.5000
9	Pulse Width	11.7000
10	Acquisition Time	0.1574
11	Acquisition Date	2019-01-07T21:12:33
12	Modification Date	2019-01-07T22:06:20
13	G to G'	1.00000000
14	Echo Delay	0.0000
15	Gradient Duration	0.0000
16	Spectrometer Frequency	(499.80, 125.67)
17	Spectral Width	(5482.5, 32679.7)
18	Lowest Frequency	(-247.2, -1955.8)
19	Nucleus	(1H, 13C)
20	Acquired Size	(512, 244)
21	Spectral Size	(512, 512)

Figure S30. NOESY (DMSO-d_6) spectrum of compound **4**.

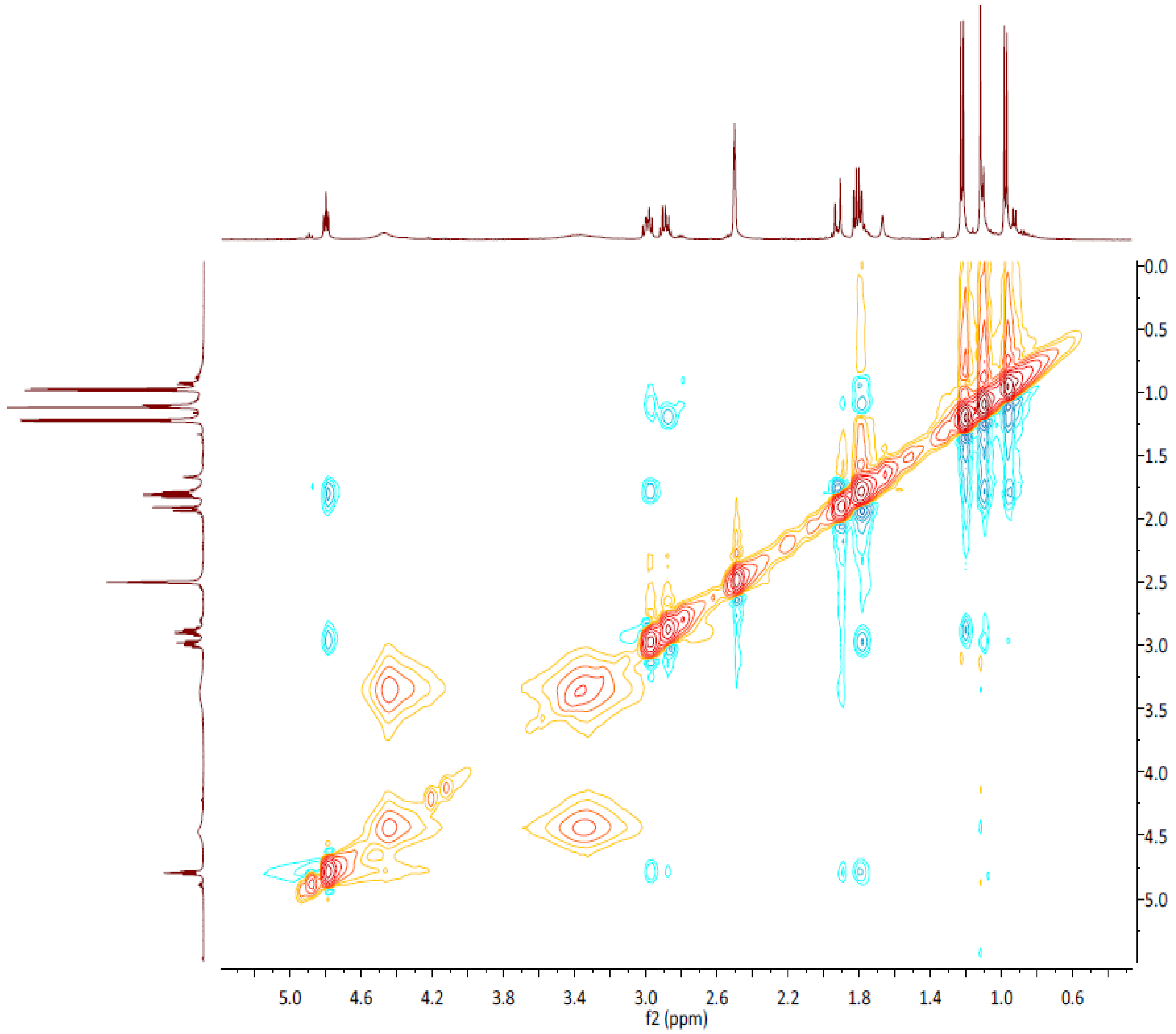

Parameters		
	Parameter	Value (f2, f1)
1	Owner	root
2	Spectrometer	av500
3	Temperature	299.5
4	Pulse Sequence	noesyph
5	Number of Scans	16
6	Receiver Gain	64
7	Relaxation Delay	2.0000
8	Pulse Width	11.7000
9	Acquisition Time	0.0934
10	Acquisition Date	2019-01-07T22:07:44
11	Modification Date	2019-01-08T00:23:13
12	G to G’	1.00000000
13	Echo Delay	0.0000
14	Gradient Duration	0.0000
15	Spectrometer Frequency	(499.80, 499.80)
16	Spectral Width	(5482.5, 5497.9)
17	Lowest Frequency	(-246.9, -254.6)
18	Nucleus	(1H, 1H)
19	Acquired Size	(512, 170)
20	Spectral Size	(512, 512)

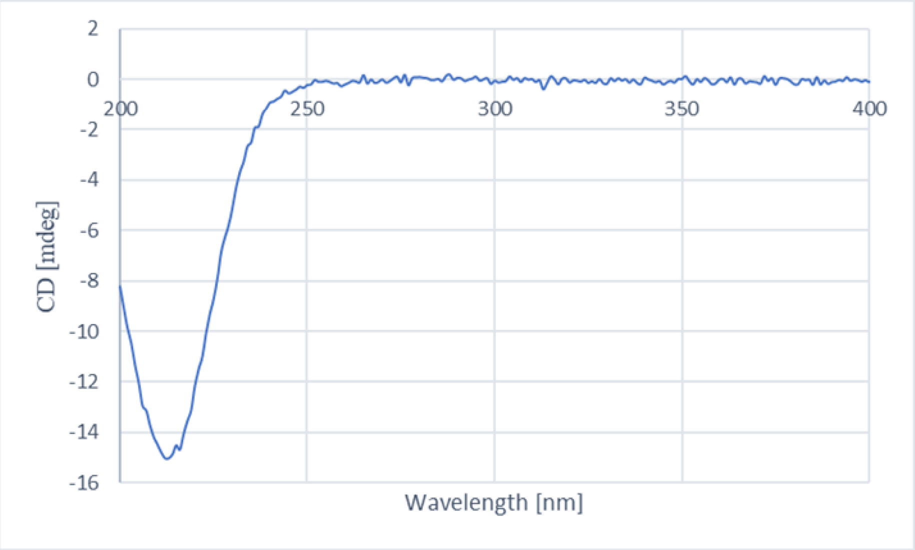

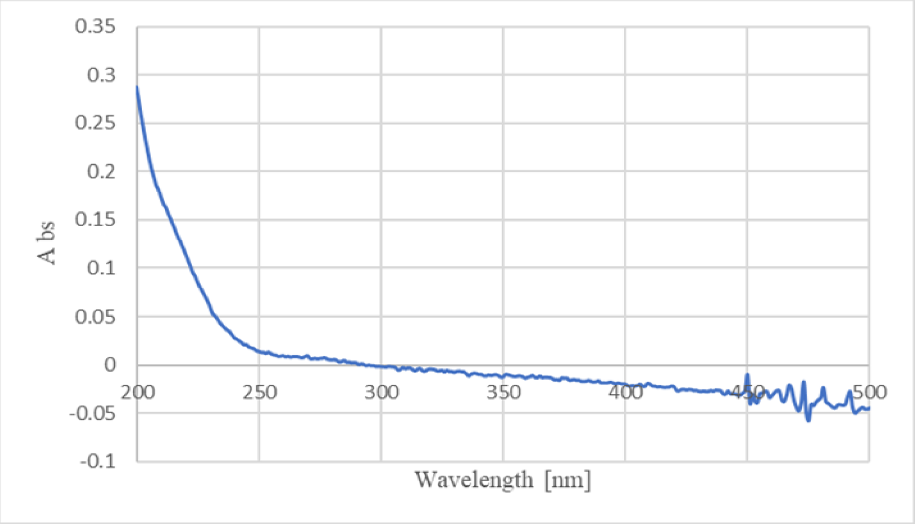

Figure S31. ECD and UV spectra of compound **4**.

Figure S32. ¹H NMR (500 MHz, DMSO-d_6) spectrum of compound **5**.

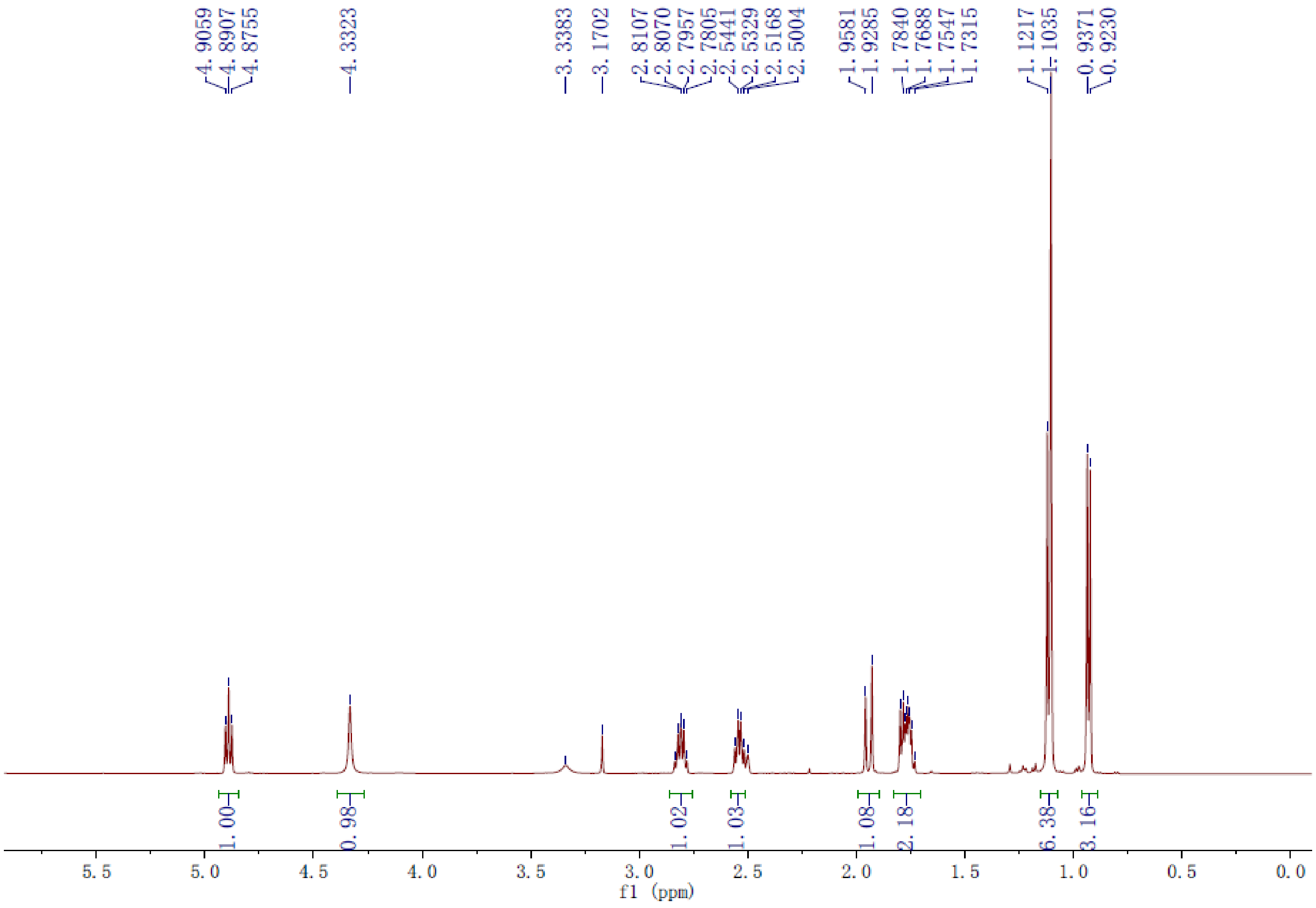

Figure S33. ^{13}C NMR (125 MHz, DMSO-d_6) and DEPT spectra of compound **5**.

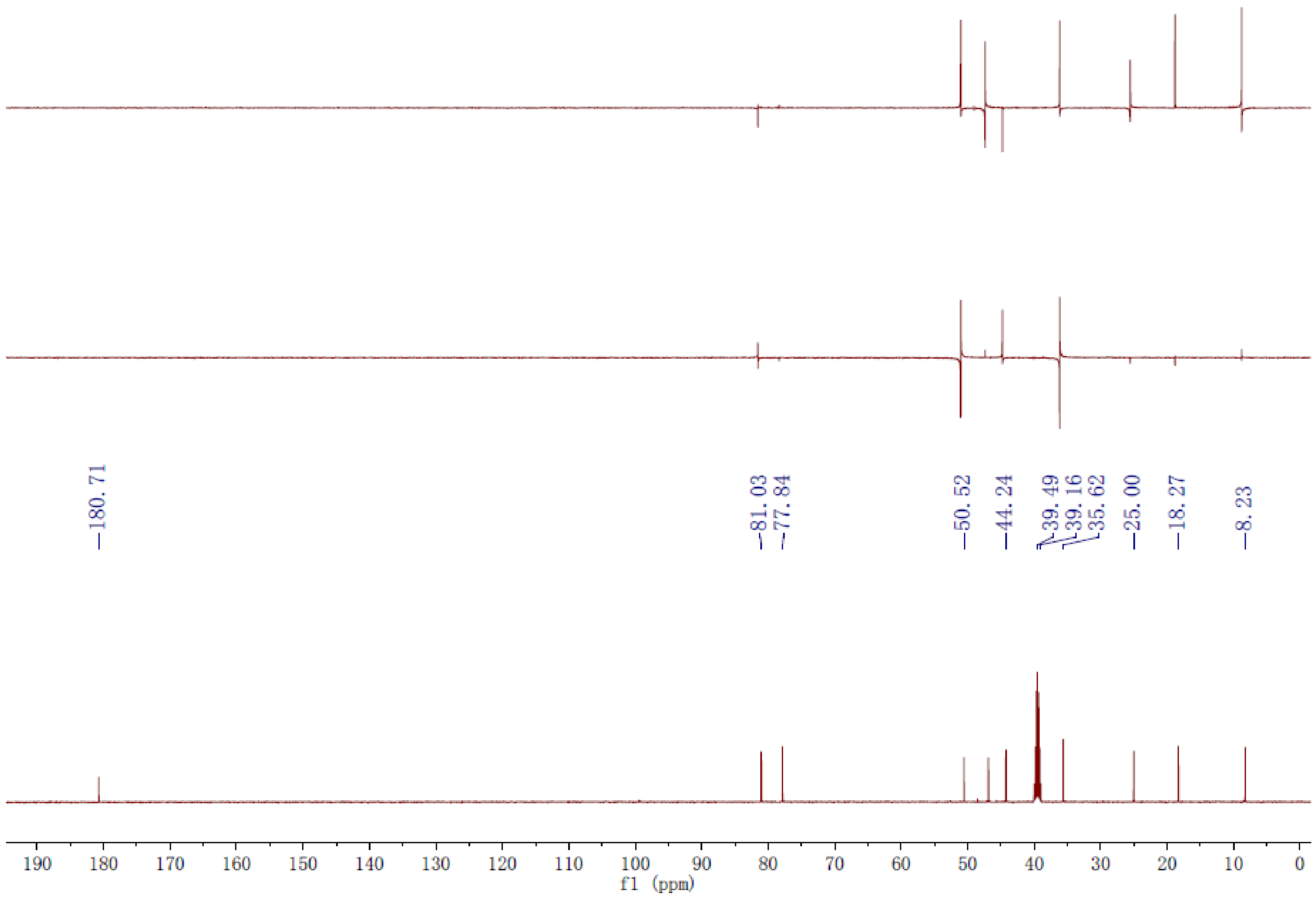

Figure S34. NOESY (DMSO-d_6) spectrum of compound **5**.

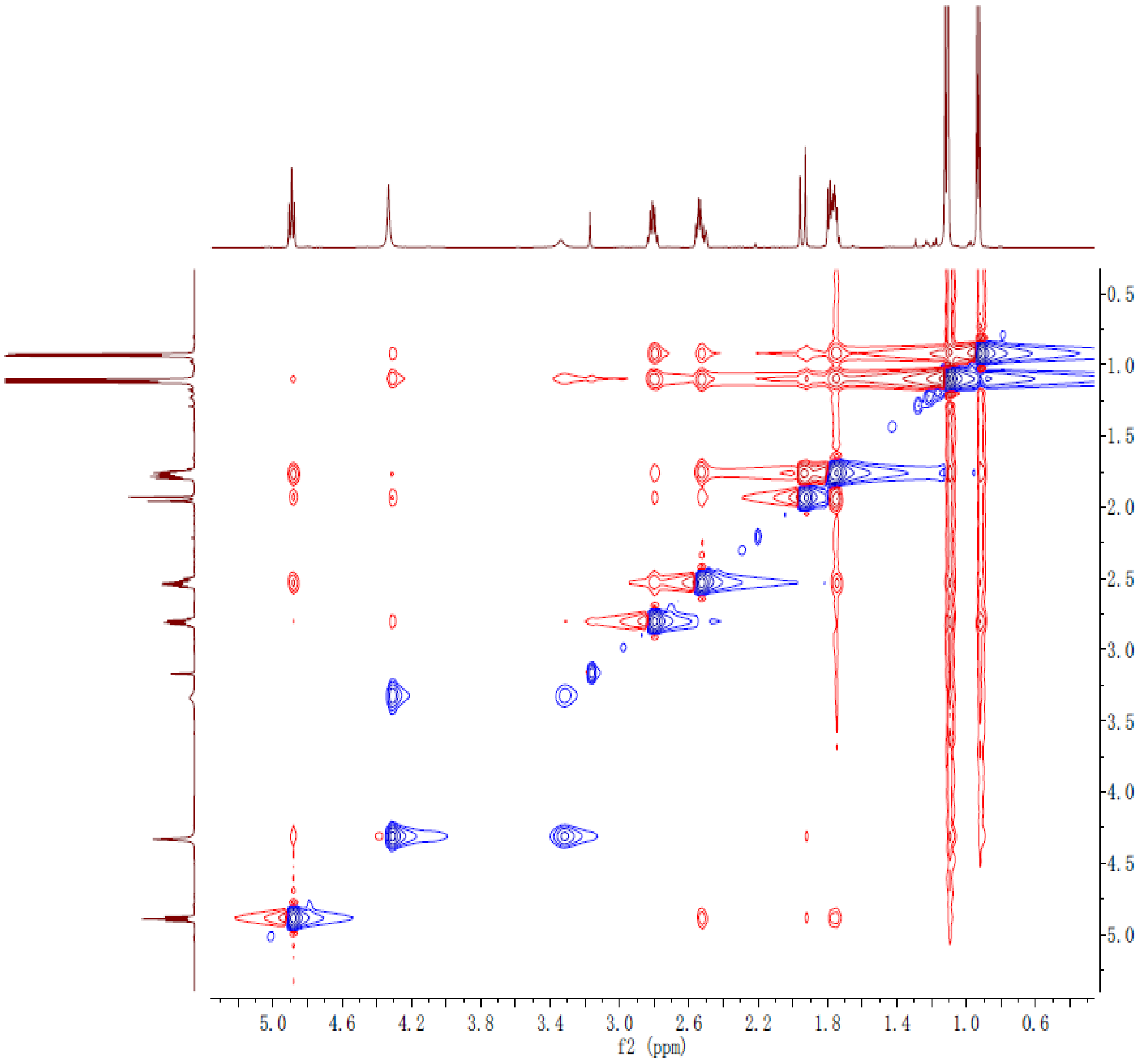

Parameters		
	Parameter	Value (f2, f1)
1	Comment	
2	Owner	root
3	Spectrometer	av500
4	Solvent	DMSO
5	Temperature	301.0
6	Pulse Sequence	noesyph
7	Number of Scans	16
8	Receiver Gain	64
9	Relaxation Delay	2.0000
10	Pulse Width	11.7000
11	Acquisition Time	0.1139
12	Acquisition Date	2017-08-03T17:49:39
13	Modification Date	2017-08-03T14:39:18
14	G to G'	1.00000000
15	Echo Delay	0.0000
16	Gradient Duration	0.0000
17	Spectrometer Frequency	(499.80, 499.80)
18	Spectral Width	(4496.4, 4498.2)
19	Lowest Frequency	(-253.7, -254.5)
20	Nucleus	(1H, 1H)
21	Acquired Size	(512, 210)
22	Spectral Size	(512, 512)

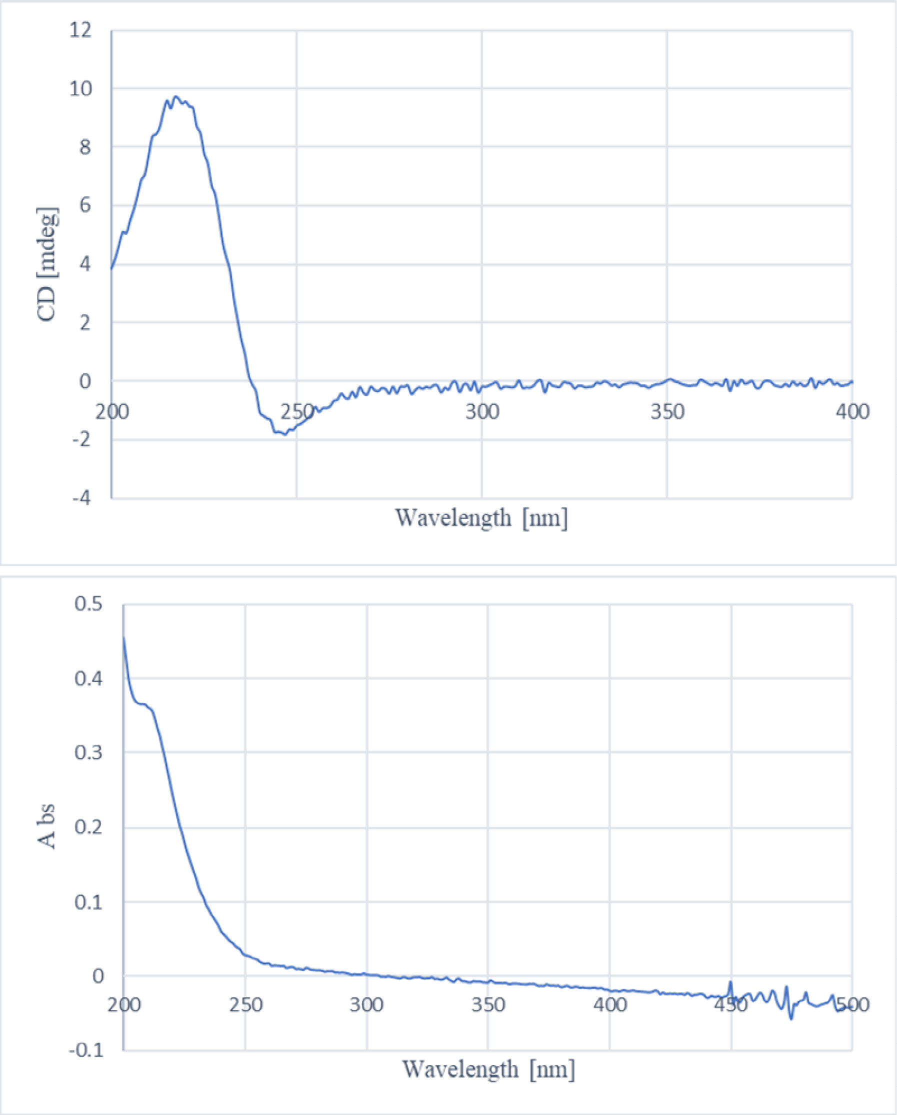

Figure S35. ECD and UV spectra of compound **5**.

Table S1. Abbreviation list.	
**Abbreviation**	**Full title**
CC	column chromatography
CE	cotton effect
DEPT	distortionless enhancement by polarization transfer
ECD	electronic circular dichroism
^{1}H-^{1}H COSY	^{1}H-^{1}H correlated spectroscopy
HMBC	(^{1}H-detected) heteronuclear multiple-bond correlation
HPLC	high performance liquid chromatography
HRESIMS	high resolution electrospray ionization mass spectrometry
HSQC	(^{1}H-detected) heteronuclear multiple-quantum coherence
MIC	minimal inhibitory concentration
MM	molecular mechanics
NMR	nuclear magnetic resonance spectorscopy
NOESY	nuclear Overhauser effect spectroscopy
PCM	polarizable continuum model
TDDFT	time dependent density functional theory
TLC	thin-layer chromatography
UV	ultraviolet
VLC	vacuum liquid chromatography
